# A multi-kingdom metabarcoding study on cattle grazing Alpine pastures discloses intra-seasonal shifts in plant selection and faecal microbiota

**DOI:** 10.1038/s41598-020-79474-w

**Published:** 2021-01-13

**Authors:** Fabio Palumbo, Andrea Squartini, Gianni Barcaccia, Stefano Macolino, Cristina Pornaro, Massimo Pindo, Enrico Sturaro, Maurizio Ramanzin

**Affiliations:** 1 Department of Agronomy Food Natural Resources Animals and Environment (DAFNAE), University of Padova, Campus of Agripolis, Viale dell’Università 16, 35020 Legnaro, Padova Italy; 2 Research and Innovation Centre, Fondazione Edmund Mach (FEM), Via Mach 1, S. Michele All’Adige, 38010 Trento, Italy

**Keywords:** Ecology, Microbiology, Molecular biology

## Abstract

Diet selection by grazing livestock may affect animal performance as well as the biodiversity of grazed areas. Recent DNA barcoding techniques allow to assess dietary plant composition in faecal samples, which may be additionally integrated by the description of gut microbiota. In this high throughput metabarcoding study, we investigated the diversity of plant, fungal and bacterial taxa in faecal samples of lactating cows of two breeds grazing an Alpine semi-natural grassland during summer. The estimated plant composition of the diet comprised 67 genera and 39 species, which varied remarkably during summer, suggesting a decline of the diet forage value with the advancing of the vegetative season. The fungal community included Neocallimastigomycota gut symbionts, but also Ascomycota and Basidiomycota plant parasite and coprophilous taxa, likely ingested during grazing. The proportion of ingested fungi was remarkably higher than in other studies, and varied during summer, although less than that observed for plants. Some variation related to breed was also detected. The gut bacterial taxa remained stable through the summer but displayed a breed-specific composition. The study provided insights in the reciprocal organisms’ interactions affecting, and being affected by, the foraging behaviour: plants showed a high temporal variation, fungi a smaller one, while bacteria had practically none; conversely, the same kingdoms showed the opposite gradient of variation as respect to the animal host breed, as bacteria revealed to be the group mostly characterized by host-specificity.

## Introduction

Quantifying the botanical composition of the diet selected by free-roaming herbivores has a long history and has been studied for several purposes. In behavioural ecology, variation of diet selection in response to seasonal and spatial heterogeneity of feed resources^1,2^, individual features^3,4^ and population density^5,6^ is a key component of foraging ecology, which determines the acquisition of trophic resources and consequently may impact on individual fitness^7^ and population dynamics^8^. In conservation ecology, diet selection is studied to investigate how foraging behaviour contributes to sustaining or degrading natural communities and biodiversity^9,10^ and influences the interactions between herbivore species^11,12^. In grassland-based livestock farming systems, understanding diet selection and its variation in response to grazing practices, as for instance stocking density^13^, livestock species, breed or category^14–16^, and supplementary feeding^17^, could help optimising the management to ensure animal productivity while conserving the sward desired composition, biodiversity and feeding value^18^. Moreover, diet selection is interesting because specific compounds present in ingested plants can be transferred directly or indirectly (if new compounds are produced by the microbiota) to animal products, such as milk or meat, in this way influencing nutritional and organoleptic properties^19,20^.

Techniques used to evaluate diet botanical composition can be direct, through video recording systems and/or behavioural observations^21^, or more frequently indirect, based on collection and analysis of ruminal or faecal samples with micro-histological techniques^22,23^, stable isotopes^24^, spectral characterization through near-infrared reflectance spectroscopy^25^, analysis of plant cuticle wax alkanes^26^, and DNA barcoding. This last method proved to be one of the most accurate and versatile for characterizing diet composition^27^, especially when the diet cannot be defined morphologically^22^. It relies on the sequencing of target DNA fragments from the residues (mainly in the animal faeces) and on the matching to a database of known sequences to identify the taxonomic origin^12,28,29^. This technique, thanks to the advent of next generation sequencing platforms, offers fast processing and turnaround times, with a very low risk of subjective interpretation. Thus, DNA-based studies have examined the botanical composition of diets selected by domestic and wild ruminants, as for example sheep (*Ovis aries*)^28,29^, goat (*Capra hircus*)^30^, white-tailed deer (*Odocoileus virginianus*)^9^, and American bison (*Bos bison*)^31^, or the dietary niche overlap between different species^12,30,32^. Additionally, the main advantage that makes this technique very attractive is that it allows investigating whatever mix of biological entities, including plant, bacterial and fungal taxa. In fact, using kingdom-specific primer pairs, it is virtually possible to recognize all the taxa composing a complex matrix. The *rbcL*, *matK*, *trnL* loci, alone or in combination, are largely applied for plants^27,29,33^, while the bacteria kingdom is almost exclusively investigated through the 16S rRNA region sequencing^34^, and the Internal Transcribed Spacer (ITS) is commonly used for fungal identification^35^.

Rumen bacteria and fungi are major components of the highly diverse microbial ecosystem that enables ruminants to efficiently utilize forage sources rich in cell wall components, and delivers other important services for the nutrition and health of the hosts^36–38^. Therefore, increasing the knowledge on gut microbiota composition in relation to dietary variations is an essential step to understand the efficiency of animal-feed interactions of ruminants^39^. Recent studies have addressed the core microbiota composition and co-occurrence of microbial and fungal taxa in the gut of cattle and other herbivorous taxa^40–45^, or investigated qualitative and quantitative changes in gut microbiota following shifts from dried forage to mixed forage-concentrate diets in domestic goats^46^ and cows^47^ or from non-grazing to grazing diets in sheep^48^, or the relationship between microbiota composition and feed efficiency in steers^49^, or addressed microbiota seasonal variations in wild ruminants^50–52^.

Despite the enormous potential of DNA barcoding, published studies using this approach to examine diet selection of grazing cattle are very few^12,53^, and we found only one study addressing also the associated covariation of gut microbiota^54^. In addition, these studies were conducted in US and African rangelands at very wide spatial and long seasonal scales, which imply remarkable variation in diet botanical composition, nutritional value and climatic conditions, that can influence gut microbiota. There is therefore a lack of knowledge on diet selection and on the possible microbiota co-variation by grazing domestic ruminants, especially in other grazing contexts. This work addresses such gap by using a DNA-barcoding approach to examine the intra-seasonal qualitative and quantitative changes in the diet botanical composition and in the gut fungal and microbial taxa of lactating cows of two different breeds grazing an Alpine semi-natural grassland. These types of grasslands are managed by extensive livestock systems throughout the European mountain areas^55–57^, where they play a fundamental role for the sustainability of livestock farming and for the conservation of mountain landscape and the associated ecosystem services^58–60^. Additionally, to our knowledge there are no published results on the simultaneous co-variation of plant, fungi and microbes in grazing ruminants. Specifically, our aims and expectations can be detailed as follows. Previous observational or indicator-based studies^13,61,62^ suggested that grazing cows are able to select a diet of a higher nutritional value than that of the standing grass biomass, but gave inconsistent results on whether this was sufficient to compensate for the decrease in grass nutritional value with the advancing of vegetation phenology during summer. Additionally, a review by Rook et al.^18^ and subsequent studies in controlled grazing conditions indicated that, in cattle, the breed has limited effects on diet selection^15,63^. We aimed at expanding on these studies by using DNA barcoding to: a) verify the richness and dynamics of diet botanical composition selected by cows during summer; b) assess whether this dynamics could be related to an intake of forage of better/poorer quality, and: c) determine whether, in a pasture highly variable in botanical composition and managed to allow animals to move over large areas, differences between breeds could be observed. We then integrated these aims by addressing the possible variation of gut microbiota through time, with the advancing of summer, and between breeds. Since the spatial and temporal scales of this study and the associated variability in nutritional and climatic factors that may influence gut microbiota were predictably much less variable than those of existing studies^12,53,54^, we expected that, if any, variation of faecal microbial and fungal communities during summer should be small. Finally, since gut microbiome can be affected by the host characteristics (and hence show differences between cattle breeds), and may even be individually inheritable^45,49,64–66^, we expected that, if this were the case also in our study, gut microbiota should have maintained a breed-specificity, irrespective of possible intra-seasonal dynamics.

From the methodological standpoint, we chose to work with faeces in light of the following considerations. Although the compositions of rumen and faecal microbiota show a clear tract-specificity, they feature a high number of co-occurring taxa^44,67–69^; moreover, faeces offer a practical and non-invasive shortcut to acquire extensive information on ruminant’s gut microbiome, which has been exploited in a number of the above cited studies^12,49,53,54^.

## Materials and mMethods

### Study site, flora characterization and faeces sampling

The followings methods were carried out in accordance with relevant guidelines and regulations.

The study was carried out during July and August 2017 on the Ombretta summer farm, located at 1900 m a.s.l. in the Dolomites, eastern Italian Alps (46.424549; 11.880871). Summer farms are temporary units were livestock is traditionally moved during summer to graze on Alpine semi-natural grasslands^70^. The grazing surfaces of Ombretta cover approximately 35 ha, with a maximum elevation of 2100 m a.s.l.

The botanical composition of pastures was investigated using a modified Braun – Blanquet method^71^. A botanical survey was performed by recording all vascular species and visually estimating their relative abundance in 65 sampling sites distributed across the grazed surface. The resulting matrix of identified plant species was subjected to hierarchical clustering with Euclidean distances and the average linkage method^72^.

During the study period the Ombretta summer farm hosted 21 lactating cows of two breeds: Simmental (14 cows) and Alpine Grey (7 cows). The stocking rate was therefore 0.6 livestock units/ha. Each morning, after the milking and at approximately 8.30- 9 am, the cows were led to graze in a different section of the pasture area, where they were kept until 12.30–1.00 p.m. In the afternoon, they were left free until they returned spontaneously to the barn for the evening milking (at approximately 6–6.30 p.m). The cows spent the night inside the building. Supplementary feeding, a common practice in many summer farms^73^ was here very low, since each cow received daily only a small amount (0.5–0.8 kg) of a compound feed (crude protein: 19.0%; crude fibre: 6.4%, total ash: 9.2%), containing also the mineral and vitamin supplement. Therefore, the cows depended essentially on grazing for fulfilling their nutritional requirements. To avoid possible contamination of samples collected from the ground^74^, individual faecal grab samples were obtained from the rectum of 9 Simmental cows and 5 Alpine Grey cows on four dates (36 samples in total, in each sampling date 9 cows were sampled; as it was not possible to sample the same cows on each date, two cows were sampled 4 times, 6 cows were sampled 3 times, 4 cows were sampled 2 times, and 2 cows were sampled once) across a two-months period (July 13th and 27th, August 21st, and September 11th, hereafter called T1, T2, T3 and T4, respectively), while the cows were tied in the barn before the evening milking, and stored at – 20 °C within three hours. This experimental protocol was approved by the Ethical Committee of University of Padova “Organismo preposto al benessere animale”, Protocol No. 42/2017.

Milk production of sampled cows was low and comparable between breeds (GLM least square means: Simmental: 11.4 kg/day, SE = 0.9; Alpine Grey: 12.9 kg/day, SE = 1.6; *p* = 0.39), Similarly, there were not remarkable differences in body size as indicated by girth circumference (GLM least square means: Simmental: 192.4 kg/day, SE = 0.9; Alpine Grey: 184.3 kg/day, SE = 1.6; *p* = 0.19).

### Metabarcoding analyses

Total DNA was extracted from the 36 faecal samples using the PowerSoil DNA isolation kit (MO BIO Laboratories Inc., CA, USA) according to the manufacturer’s instructions.

Plant species identification was achieved by sequencing the chloroplast *trnL* (UAA) intron region using the primers A49325: 5′ CGAAATCGGTAGACGCTACG3 3′and B49466: 5′ CCATTGAGTCTCTGCACCTATC 3′^75^ with specific overhang Illumina adapters for the amplicon library construction. Each sample was amplified in a 25 µl PCR reaction with 5 µl of 5X Flexi Buffer (Promega, Inc., Madison, WI, USA) 0.125 µl of GoTaq DNA Polymerase (5u/μl, Promega, Inc.), 1 µl of forward and reverse primers (10 µM) and 2.5 µl of template DNA (5–20 ng/µl). PCR reactions were executed with GeneAmp PCR System 9700 (Thermo Fisher Scientific, Pittsburgh, PA, USA) with the following cycling conditions: initial denaturation step at 95 °C for 2 min; 40 cycles at 95 °C for 15 s, 52 °C for 15 s, 72 °C for 30 s; final extension step at 72 °C for 5 min.

The fungal component was investigated through the amplification of a portion of the ITS region, while a fragment of the 16S rDNA gene was used for identification of bacteria. In both cases, primer couples, indexing and libraries preparation followed the procedure described in Coller et al.^76^. Sequencing was carried out on an Illumina MiSeq (PE300, Illumina, Inc., San Diego, California, U.S.) platform (MiSeq Control Software 2.5.0.5 and Real-Time Analysis software 1.18.54.0, both provided by Illumina, Inc.).

Sequencing and bioinformatics analyses for plants, fungi and bacteria were performed according to Coller et al.^76^, while Operational Taxonomic Units (OTUs) assignment was performed using the MICCA pipeline proposed by Albanese et al.^77^.

Specifically, for plant-deriving reads, OTUs were clustered with 99% similarity cut-off. OTUs characterized by chimera sequences > 200 bp and/or by < 360 reads (i.e., on average, less than 10 reads per sample^30^ were excluded from further analyses. Considering that *trnL* sequences are not available in the BOLD System database and that GenBank is a sequence repository that could be more prone to the presence of erroneous data^78^, the remaining OTUs classification was performed through a manually curated NCBI BLAST search, based on the Megablast algorithm (E-value ≤ 1e−50, query coverage > 95%) and on the GenBank nucleotide collection (nr/nt) used as database. The following criteria were adopted^32,74,79^: (i) family name was assigned when the best identity score was ≥ 0.96; (ii) species or genus names were accepted if the best identity score with a query OTU was ≥ 0.98; (iii) if two or more species (e.g. *Ranunculus acris* L., *Ranunculus montanus* Willd.) were assigned to a given OTU with the same match score, we assigned the OTU to the lowest taxonomic level (genus, i.e. *Ranunculus*). Those OTUs whose identity scores resulted < 0.96 were considered as “unclassified” and excluded from the subsequent analyses.

Instead, for fungi and bacteria-deriving reads, OTUs were clustered at 97% similarity cut-off and taxonomic prediction (or OTUs assignment) were performed according to Coller et al.^76^. The assignment was based on the RDP classifier v.2.11 (https://rdp.cme.msu.edu/)^80^ and the UNITE database (https://unite.ut.ee/#main).

The efficiency of our sampling structure in terms of plants, fungi and bacteria richness was evaluated analysing the cumulated number of taxa detected against the number of individuals collected using accumulation models^79^ and rarefaction analyses were performed according to Albanese et al.^77^. The rarefaction curves for plants, fungi and bacteria are shown in Supplementary Fig. S2, S3 and S4.

### Data analysis

Molecular data from the three kingdoms (plants, fungi and bacteria) were analysed separately using Calypso^81^. The relative abundances of taxa within kingdoms (plants, fungi and bacteria) were normalised by applying a total sum scaling (TSS) normalisation followed by square route transformation.

Complex associations between the relative abundances of genera/species of plants, fungi and bacteria were then examined, within each kingdom, according to the variables “collection time” (i.e. the four different sampling dates T1, T2, T3 and T4) and “breed” (i.e. Simmental and Alpine Grey), with principal coordinate analysis (PCoA) using the Bray–Curtis similarity index^82^, principal component analysis (PCA), canonical correspondence analysis (CCA) and redundancy analysis (RDA). We used these four independent ordination approaches to ensure wide support to the ensuing inferences.

The alpha diversity of plants, fungi and bacteria was measured using three ecological indexes: i) the richness index (R), that simply considers the number of present taxa/OTUs; ii) the Pielou's evenness index^83^ (J), that measures how evenly abundant the present taxa/OTUs are; iii) the Shannon index^84^ (H) to evaluate the overall diversity considering both richness and evenness.

To examine whether the relative abundance of individual taxa varied with collection time and between breeds, we fitted linear mixed effect regression models with the individual cow as random effect. P-values were adjusted by Bonferroni correction and False Discovery Rate.

Finally, we associated to each plant taxon identified an index of forage value (Supplementary Table S1) varying from -1 (harmful) to 8 (high quality), which is used in pasture management as a common proxy for the nutritive value and palatability of single plant species^85,86^. Based on the relative faecal abundance of each plant taxon and the corresponding index of forage value, we calculated for each faecal sample a weighted average “forage value”. After log-transformation to obtain a gaussian distribution, we then analysed these values with a simple linear mixed model using the *lme* package in R^87^ with the fixed effect of period and the random effect of individual.

## Results

### Plant composition of grazed surface

The cluster analysis of identified plant taxa yielded 6 groups (Supplementary Fig. S1). Species abundance, averaged within group, is reported in Supplementary Table S1. Two groups included only two surveys each and described an area nearby the stable. Group 1 was characterised by a great abundance of nitrophilous species such as *Urtica dioica*, while surveys included in group 4 were dominated by *Deschampsia caespitosa* and *Ranunculus thora*. Groups 2 and 3 included surveys with more than 20% of *Sesleria varia*. In group 2 *Carex caryophyllea*, *Homogyne alpina*, and *Stachys alopecuros* were also highly abundant, while in group 3 *S. varia* was accompanied by *Petasites albus*, *Polygala alpestris*, *Pulsatilla alpina*, and *Tussilago farfara*. Surveys included in group 5 were characterised by the presence of *Festuca violacea*, *Agrostis stolonifera*, and *Anthoxanthum odoratum*, while group 6 by *Festuca rubra* and *S. varia*. About 50% of the grazed area was included in group 6; groups 3, 5, and 2 were represented respectively for 24, 13, and 7%.

### Metabarcoding-based plant taxa abundance and diversity

The next-generation sequencing of the *trnL* P6 loop region produced 1,839,794 reads. After demultiplexing, trimming, merging and sequence filtering, 1,731,461 reads were obtained, and each sample was represented, on average, by 48,096 (± 9,556) reads. Overall, 17,470 OTUs were detected based on 99% nucleotide sequence identity between reads. Rarefaction curves showed a suitable homogeneity across samples with an output between 2472 and 4528 OTUs stemming from a sequencing depth ranging from 30,012 to 71,090 (Supplementary Fig. S2). Of these OTUs, 278 (11,052 reads, 0.6% of the total) were removed because characterized by chimera sequences ranging from 200 to 400 bp, while 16,737 OTUs (353,325 reads, 20.4% of the total) were removed because they contained less than 320 reads (i.e., on average, less than 10 reads per sample^30^). The remaining 455 OTUs accounted for 79.0% of the total reads. Due to the complexity of a manually curated NCBI BLAST search, we restricted the OTU classification to the 200 most abundant OTUs that represented 72.7% of the total reads. Despite this, the average sequencing depth per sample remained as high as ~ 35,000 reads (Supplementary Table S2).

**Figure 1:**
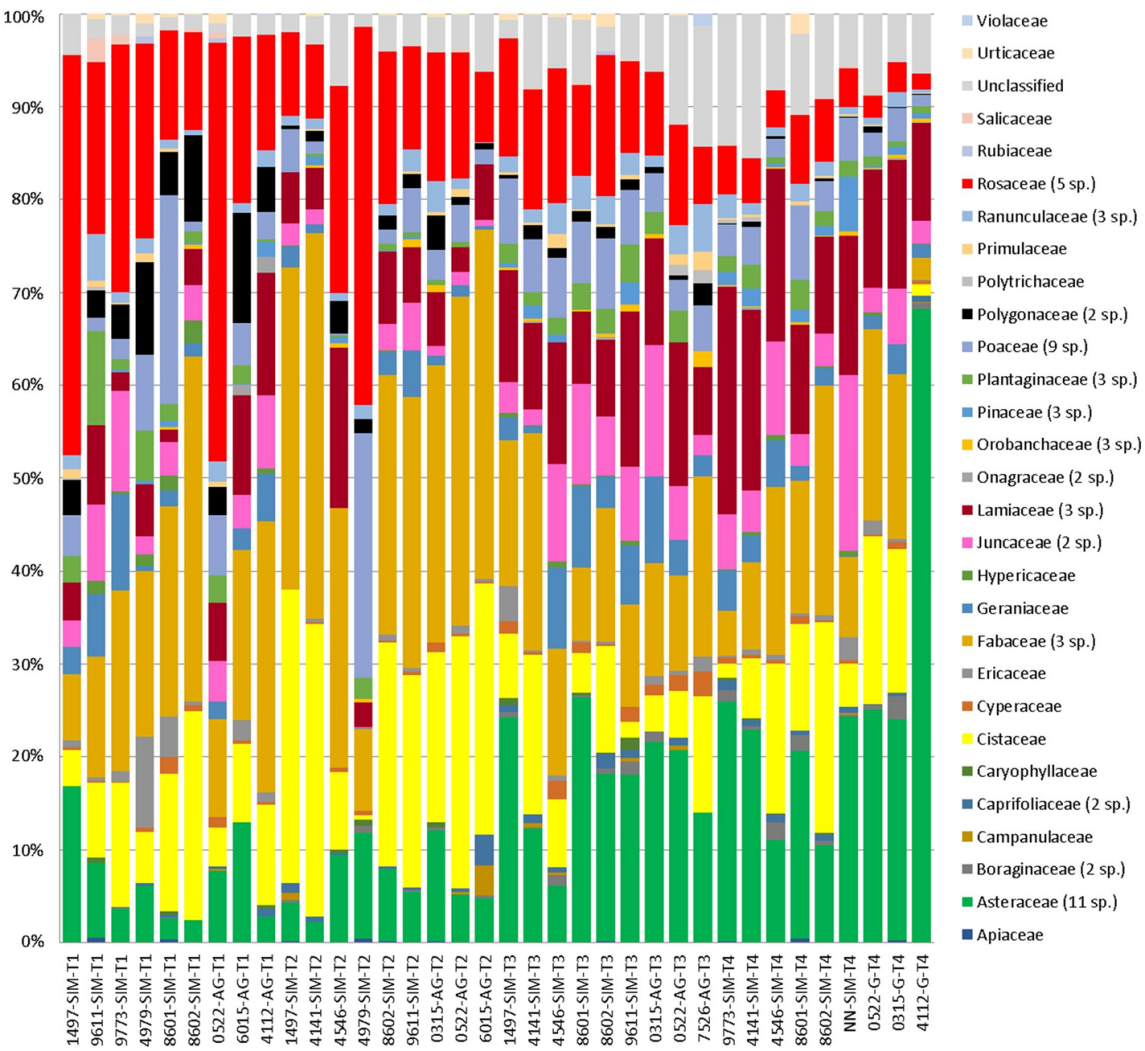
Clustered bar chart of the relative percent abundance showing the substitutional shifts of the plant taxa, grouped at family level, identified by DNA sequencing from bovine fecal material. The number of species featured by families in which more than one taxon was found is indicated in brackets. Samples belonging to the four time points (T1 trough T4) are ordered from left to right and coded by cow individual numbers and breed (SIM: Simmental; AG: Alpine Grey).

According to the BLAST results, 99.9% of the reads were distributed (identity score ≥ 0.96) among 30 families (see Fig. 1 for a visual representation, and Supplementary Table S2 for the list of taxa). Among these, the five most abundant were Cistaceae (20.5%), Asteraceae (18.1%), Rosaceae (17.2%), Lamiaceae (12.4%), and Poaceae (6.5%) that accounted for 74.6% of the total abundance, while other 5 families, ranging in individual abundance from 6.1% (Poaceae) to 1.7% (Juncaceae) accounted for a further 16.5%. As clearly suggested by Fig. 1, families abundances varied throughout the study period. The mixed effect regression modelling indicated that, among the 10 most abundant families, Asteraceae, Lamiaceae and Juncaceae showed significant increasing trends from T1 to T4, while Rosaceace, Fabaceae, and Polygonaceae followed significant decreasing trends (Supplementary Fig. S5). Family abundance was instead always comparable between faecal samples of the two breeds, with the only exception of the Hypericaceae, quantitatively a minor portion as they accounted for 0.41% of the data, but which were significantly more abundant in the Simmental breed (*p* < 0.01).

**Figure 2:**
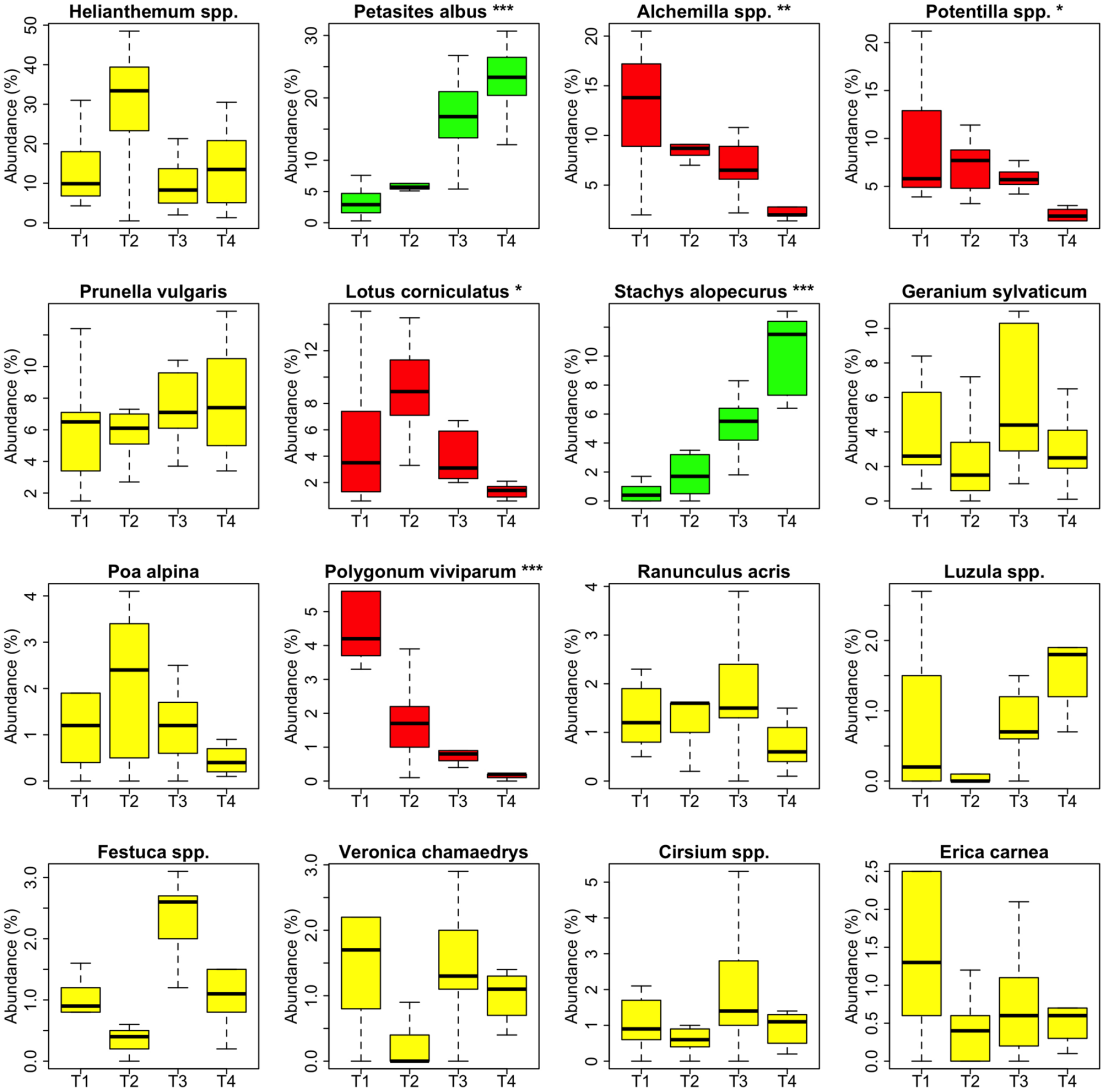
Variation of the relative abundance (% of total abundance) of the 16 plant genera/species identified in faecal samples with a relative abundance > 1% in relation with collection time (T1, T2, T3 and T4). Plant taxa are ordered from left to right and top to bottom according to decreasing average relative abundance. The significance of the effect of collection time in the linear mixed regression models analysing relative abundances (standardized with total sum scaling (TSS) normalisation followed by square route transformation) is indicated as ***P < 0.001; **P < 0.01; *P < 0.05. Boxplots in yellow indicate non-significant temporal trends, boxplots in green indicate a pattern of increase with collection time, boxplots in red indicate a pattern of decrease.

At lower taxonomic ranks, 93.1% of reads were assigned to 67 genera and 39 species (identity score ≥ 0.98) (Supplementary Table S2) Overall, each faecal sample contained from 30 to 56 genera/species (mean 41.8). Most of the identified taxa (51) showed a very low abundance (< 1%), overall accounting for 13.8% of the total. Among the other 16 taxa, abundances > 5% were registered only for 7 genera/species, with *Helianthemum* spp., *Petasites albus* and *Prunella vulgaris* being the most abundant (16.6%, 14.3% and 6.7% respectively, Supplementary Table S2). Significant variations in relative abundance with collection time were found for *Petasites albus* and *Stachys alopecuros*, that increased from T1 to T4 (Fig. 2), and for *Alchemilla* spp., *Potentilla* spp., *Polygonum viviparum,* and *Lotus corniculatus*, that decreased. Noteworthy, five of them (i.e. *Petasites albus*, *Alchemilla* spp., *Potentilla* spp., *Lotus corniculatus*, *Stachys alopecuros*) were also among the ten most abundant taxa (Supplementary Table S2). As observed for families, there were no major differences in taxa relative abundances between breeds, except for *Hypericum perforatum*, which was the only member of the Hypericaceae family, whose presence was higher in the Simmental cows as mentioned above.

**Figure 3:**
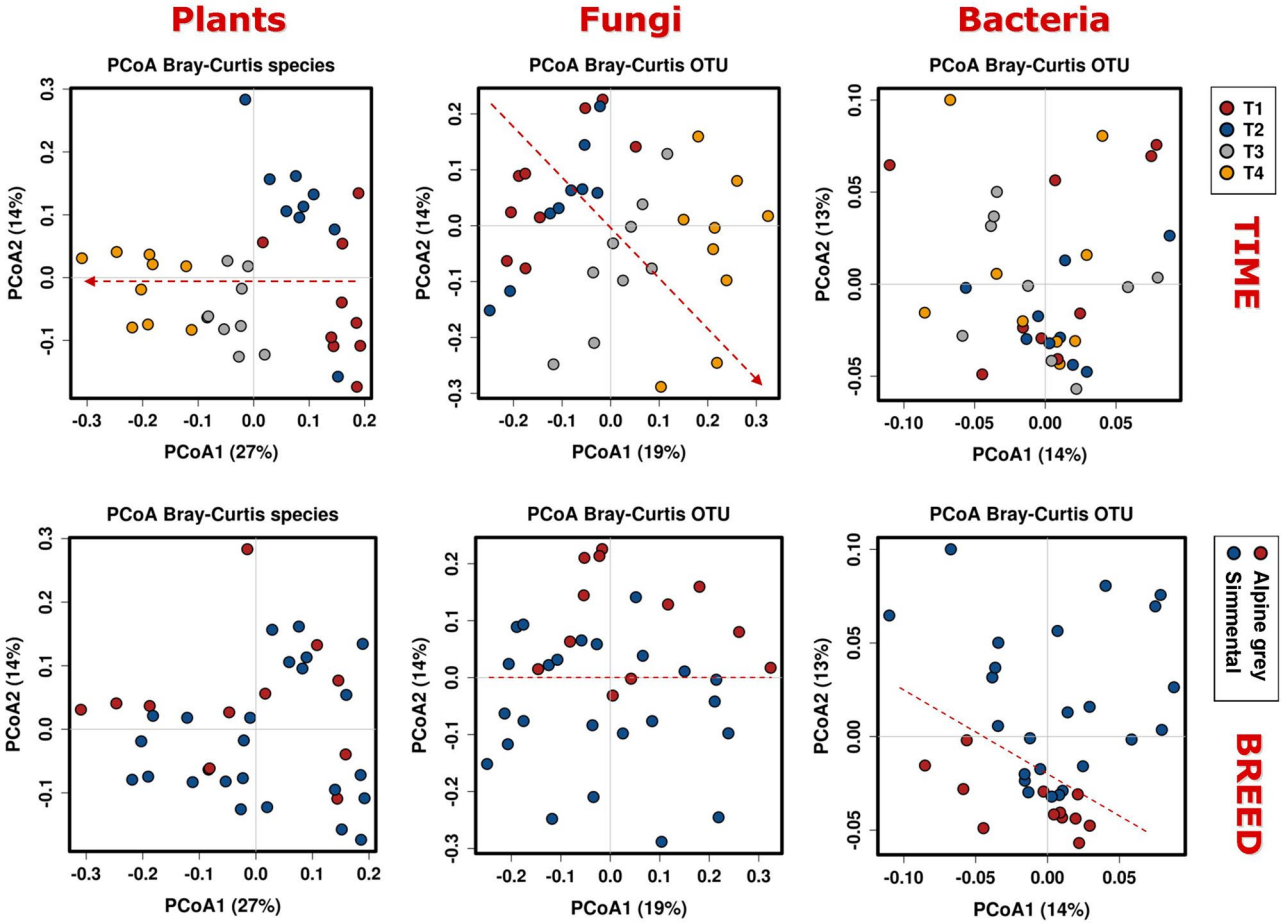
Principal coordinate analysis showing synoptically the ordination plots of standardized relative abundances of plant, fungal and bacterial taxa for the variables “collection time” and “breed”. Dotted arrows indicate gradients of time-consistent data partitioning. Dotted lines divide parts of the quadrants fitting a breed-consistent separation.

In order to examine the significance of all data at family or genus/species level, and to compare them also with the ones from the fungal and bacterial surveys (see below), sequencing data were subjected to multivariate analyses. At the plant genus/species level, the Bray–Curtis index-based PCoA plot (Fig. 3, left panels) revealed that most of the samples clustered according to collection time. The first axis, which explained 27% of the total variance, was able to separate the T1-T2 samples from the T3-T4 samples (Fig. 3, upper left panel). The same analysis did not indicate a clear clustering according to breed (Fig. 3, bottom left panel).

**Figure 4:**
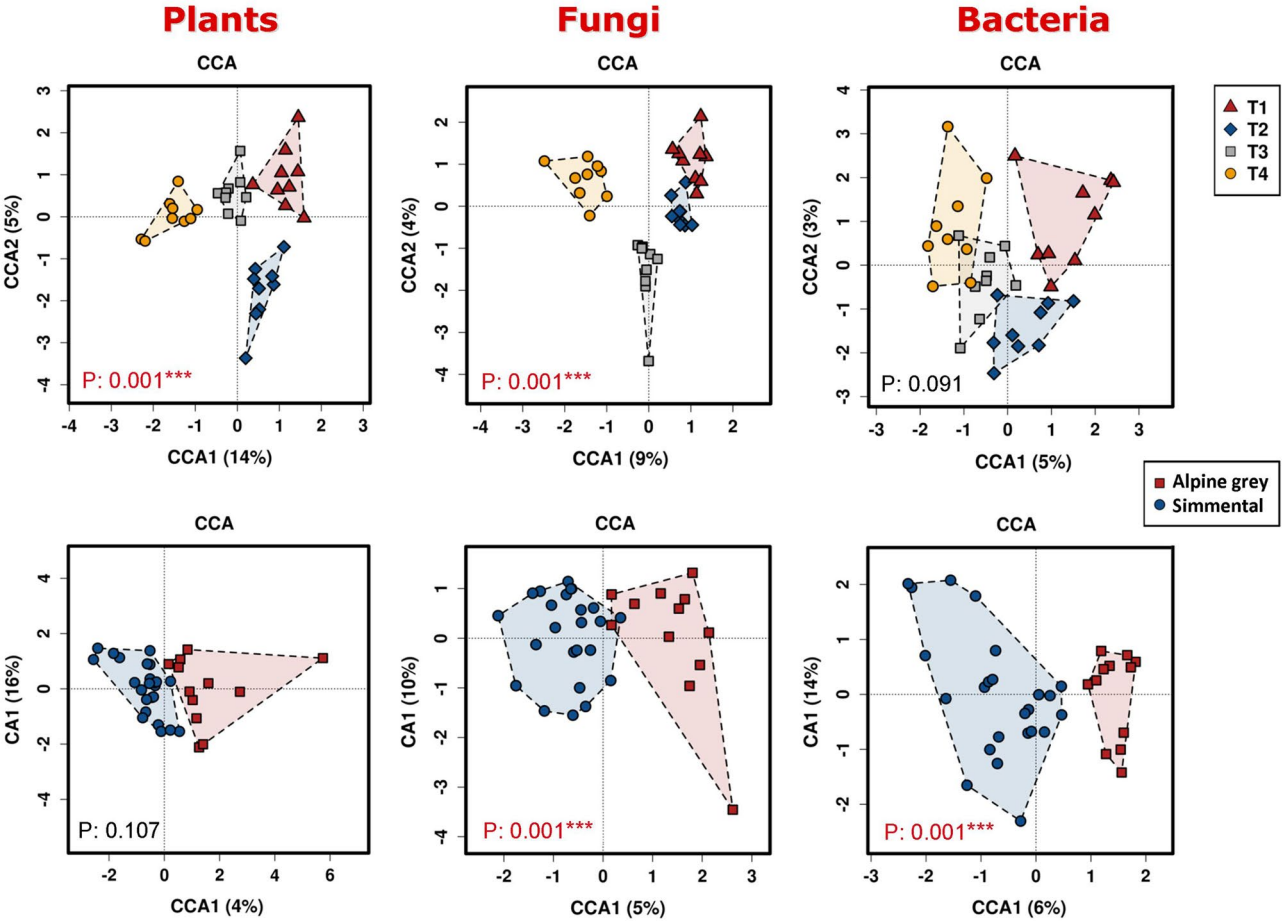
Canonical coordinate analysis of standardized relative abundances of plant, fungal and bacterial taxa in relation to sampling time and cow breed. The respective significance levels are indicated into the panels and highlighted in red when significant *p* values were obtained.

The statistical significance of the PCA clustering was also confirmed by the canonical correspondence analysis (Fig. 4, upper left panel): collection time significantly explained variations observed among plant species (Chi square = 0.18; F = 3.18; *p* = 0.001), while breed did not (Chi square = 0.03; F = 1.28; *p* = 0.107) (Fig. 4, bottom left panel).

Similar results were obtained with the Euclidean distances-based PCA (Supplementary Fig. S6): along the PC1 axis the T1–T4 faecal samples groups showed a small average intra-cluster distance, revealing a substantial similarity among samples collected on the same date, while the analysis according to breed showed a large average intra-cluster distance (and thus a great variability in terms of diet composition within each breed) and a marked overlap between the Simmental and Alpine Grey diets. Finally, also the Redundancy Analysis indicated a highly significant effect of collection time (Supplementary Fig. S7), and no effect of breed (Supplementary Fig. S8) on the variations of relative abundances of plant taxa.

Among the ecological indexes used to examine the plant diversity, the richness index increased gradually and significantly from T1 to T4 (Supplementary Fig. S9), with an average number of plant genera/species ranging from 37.0 (T1) to 49.6 (T4, Supplementary Table S2). This finding was also in agreement with the number of exclusive genera/species (i.e. time point specific taxa) detected: T1 and T2 did not show any exclusive taxa, whereas at T3 and T4 two and six time point-specific taxa were identified, respectively (Supplementary Fig. S10). The Evenness and Shannon indexes did not follow such a clear temporal pattern, being lower at T2 and T4 (*p* < 0.001 for both indexes), indicating that in these two time points some plant taxa were meaningfully more abundant than others (Supplementary Fig. S9). The same ecological diversity indexes were comparable between the two breeds, except for the Richness index, that was higher (*p* = 0.028) in the Simmental samples (Supplementary Fig. S11). In fact, the faecal samples of this species contained on average 43.5 different genera/species, and those of Alpine Grey 38.3 (Supplementary Table S2). This is also reflected in the number of exclusive taxa (i.e. breed specific taxa) characterizing the two breeds: 1 for Alpine Grey and 10 for Simmental (Supplementary Fig. S10). The Index of forage value calculated for forage samples showed a clear temporal trend, declining in T3 (*p* < 0.01), and further in T4 (*p* < 0.001), as respect to T1 and T2 (Supplementary Fig. S12).

### Fungal diversity

The sequencing of the ITS fungal region yielded 2,140,615 filtered reads. Upon processing and OTU clustering, 1,621 OTUs were identified (Supplementary Fig. S3). The whole dataset list of identified fungi is available as Supplementary Table S3 while the 25 most abundant taxa are graphically represented in Supplementary Fig. S13. The phylum Ascomycota dominated, representing 52% of the OTUs, mainly with the classes Dothideomycetes (19.6%), Sordariomycetes (10.9% and Pezizomycetes (7.4%). The second most represented phylum was the rumen symbionts Neocalligasticomyocta (21.1%), with the only class Neocallimastigomycetes, followed by Basidiomycota (14.8%).

Similarly to what observed with plants, although less markedly, a time-related variation in the composition of the fungal community was observed. This can be appreciated visually, for the first 25 most abundant OTUs which encompass > 65% of the abundance, in Supplementary Fig. S13. The mixed model analysis indicated significant temporal trends for 11 taxa, 7 of which were within the 25 most abundant (Supplementary Fig. S14). Increasing trends were observed for the Ascomycota genus *Camarosporium*, which accounted for more than 10% of total OTUs in the second half of the summer, and for the Basydiomicota *Ustilago striiformis,* although with a much lower final abundance (3%). Decreasing trends were observed instead for the Ascomycota family Pyronemataceae, genus *Sporormiella*, species *Podospora myriaspora* and *Ustilago striiformis*, and for an unidentified Ascomycota OTU.

Considering all taxa, the temporal variation is confirmed by the PCoA analysis (Fig. 3), further supported by the CCA (Fig. 4; effect of collection time: Chi Square = 0.24; F = 1.93; *p* = 0.01) and by the PCA and RDA (Supplementary Fig. S6 and S7). In contrast with plants, instead, the multivariate analyses found that fungi displayed a certain specificity for breeds (see Figs. 3 and 4 and Supplementary Fig. S6 and S7).

The richness index of fungi did not change with collection time, while both the Evenness and the Shannon indexes increased steadily from T1 to T4 (Fig. S9; Effect of collection time: *p* < 0.001 for both indexes), thus indicating a progressively more homogenous distribution of taxa abundances. Similar to what observed for plant, the Richness index was higher (Supplementary Fig. S11) in Simmental than in Alpine Grey samples (*p* < 0.001).

### Bacterial diversity

For the 16S bacterial amplicon from a total of 2,544,712 reads, 5630 OTUs were detected (Supplementary Fig. S4) based on 97% nucleotide sequence identity between reads. The whole dataset list of identified bacteria is available as Supplementary Table S4. Firmicutes (around 45%) and Bacteroidetes (29%) were by far the dominant phyla. The identities and abundances of the first 25 most abundant OTUs of bacteria are shown in Supplementary Fig. S15. Unlike for plants and fungi, no appreciable shifts were apparent in relation to collection time, as confirmed by the multivariate analyses (Figs. 3 and 4 and Supplementary Fig. S6 and S7). Instead, the composition of the bacterial community seemed to be linked to the breed, even more than what was found for fungi (Supplementary Fig. S6 and S8). The ecological indexes did not differ across collection times and between breeds (Supplementary Fig. S9 and S11).

## Discussion

In this DNA metabarcoding-based study, we investigated the qualitative and quantitative changes of plant taxa and fungal and bacterial communities in the faeces of cows of two breeds grazing an Alpine pasture during a two-month period.

We found that, despite the short period of two months during summer, the botanical composition of the diet showed remarkable variations with sampling time and suggested no differences in selectivity between breeds. Also the fungal community varied with time, but this was only in apparent contrast with our expectation because this pattern was mostly attributable to ingested taxa, which also apparently explained the small difference between breeds. The bacterial community, instead, showed a substantial temporal stability but a certain degree of breed specificity, in line with our predictions. In the following part, we will first discuss the variation of the single kingdoms and conclude by commenting the differences in their patterns.

With the vegetation surveys we recorded a total of 157 vascular species in the whole grazed surface of Ombretta, with a maximum of 49 and a minimum of 23 species per survey. This number was in line with what was found in other studies involving alpine pastures at the same altitude^88,89^, while the total number of species was higher. This is expectedly due to the spatial heterogeneity within the pasture area that increased species richness^90,91^.

We identified more than 60 different plant genera/species in the diet of the grazing cows. This number of taxa is within the range found in wild and domestic ruminants grazing in a variety of habitats^50,53,92–94^ and demonstrates a richness and diversity that in grazing domestic livestock had not been detected before with micro-histology or other techniques^15,95,96^. The plant identity attribution with the metabarcoding approach is achieved with a bioinformatics procedure seeking the best alignment match with reference databases, and the actual flora of a site could not be necessarily featured in such sequence repositories. Nevertheless, the taxa identified in the faeces that were found also in vegetation surveys accounted for 98.8% of the reads (excluding reads of non-identified OTUs), and the few genera/species found in faecal samples but not in pasture had a very low relative abundance (less than 0.4%). This high number of plant taxa in the faecal samples reflected the high biodiversity of the semi-natural grassland grazed by the cows, although few taxa contributed to most of the reads: six genera/species had an average relative abundance between 5 and 16.5%, accounting for 62% of the total reads (excluding non-identified OTUs), and other 12 had an abundance between 1 and 5%, accounting for 25% of the total reads (excluding non-identified OTUs). How much closely the faecal plant composition reflects directly the botanical composition of the diet ingested is still unclear^27,97^, because the sequence abundance of *trnL* genes in faeces depends also on the density of chloroplasts in the different species and on the digestibility of their plant tissues. Given also that our botanical surveys were not intended to estimate the availability of specific plants, but rather to describe the high diversity of the pasture area, we did not attempt to calculate selection indexes by comparing faecal botanical composition with estimated availability in pasture. However, the qualitative assessment holds true, and our study indicated that in these habitats these cattle breeds are typical grazers, because herbaceous plants are almost the totality of their diet, and shrub or trees are practically absent, while in other environmental conditions other cattle breeds can select substantial amount of plants^98^. In addition, the quantitative assessment is useful for comparing dietary niche overlaps or identifying temporal dynamics^12,30,31,98^. In this regard, we found no indication of a different selectivity between the two breeds, apart from a slightly higher richness index for Simmental. However, this might be due to the larger number of samples analysed for this breed. Also, other studies have found, although with different methodological approaches, only small differences in diet selectivity between different cattle breeds at pasture^16,63^.

The most interesting result of our study was that the relative abundance of plant taxa showed a clear temporal trend, with the prevalence of the Rosaceae family in the early period and their gradual fading in parallel with the increase of Asteraceae. The former family was particularly represented by *Potentilla* spp. and *Alchemilla* spp., while the latter saw a major increase of *Petasites albus*. The changes in taxa composition were associated with a clear decline in the average “forage value” of faecal samples, indicating that in the second half of the grazing period the relative ingestion of less nutritious plant species increased. This might be due to a depletion of the more nutritious species in the pasture. If the grazing pressure of the cows was initially concentrated on these species, their biomass might have decreased progressively, given that the vegetative season is limited at this altitude and plants are unable to regrow after defoliation. This would have resulted into a decline in the average nutritive value of the pasture that the cows were apparently unable to contrast with diet selection. Such decline is suggested by data on chemical composition obtained in the same pasture, that indicate a drop in nitrogen content and an increase in cell walls and lignin contents with the advancing of summer^99^. We did not find any relation between the rank of the most abundant 25 plant taxa in the faecal samples and their index of forage value (R^2^ = 0.0002). Even with the caution suggested by the potentially different specific disappearance of plant DNA during the transit through the gut, the fact that this high variability in ranking is unrelated with forage value suggests that the cows were not selecting (at least not primarily) plant species based on their nutritive value. Mayer and Huovinen^100^ obtained similar indications and argued that cattle might select forage mostly based on plant accessibility. One such kind of plant in our study was *Petasites albus*, which has a low forage value and showed a great increase in faecal samples from the first to the last period. This species has a good abundance in the groups 3 and 6 of the pasture areas, where cows tend to spend the afternoon, and is characterized by very broad leaves, that make intake easy to cows. One local constraint that might have reduced the selectivity of the animals is also that during the night they were kept indoor and could not feed, which was not compensated by the small amount of supplementary concentrate that they received. In fact, behavioural observations (Ramanzin M., unpublished) indicate that the cows spent most of their time outdoor grazing, and very little resting and walking.

While some of previous studies^13,61^ concur with a decrease in nutritive value of the diet selected during summer, they do not indicate how this is related with changes in botanical composition. The DNA barcoding approach we used allows to obtain information on which plants are consumed and are determining changes in diet quality, which may be particularly relevant to address the management of grazing in semi-natural grasslands where both animal productivity and plant biodiversity have to be conserved^101^. Further studies are needed on the temporal evolution of grazing behaviour and diet selection, faecal indexes of diet quality^102,103^ and the evolution of chemical composition of grass, to help elucidating these questions. Additional investigations, such as the analysis of other cpDNA barcoding loci (e.g. *rbc*L and/or *trnH*-*psbA*^104^), might also be useful to improve the taxonomic resolution and to deepen the knowledge of those OTUs (6.9%) that were not assigned to any taxon.

The faecal fungal community was dominated by Ascomycota, followed by Neocallimastigomyocta (anaerobic chytridiomycetous gut fungi) and Basidiomycota. In this respect, the few studies that we found on rumen or faecal fungal communities of ruminants^48,49,105^ concur in indicating a large dominance (from more than 60% to more than 80% of the OTUs) of Neocallimastigomycota, which instead in our study accounted for little more than 20% of the OTUs. Neocallimastigaceae, the only family of Neocallimastigomycota, comprises strictly anaerobic species active in degradation of cell walls as symbionts in the rumen and in the large intestine^106–108^. Conversely, the phyla Ascomycota and Basidiomycota included various genera/species of fungi that the cows most likely ingested with the forage. These included plant pathogens as *Camarosporium* spp.^109^ (9.7% of the OTUs) and *Ustilago striliformis*^110^, *Entyloma microsporum*^111^, *Microbotrium silybum*^112^, accounting overall for a further 4.4% of the OTUs, and coprophila fungi as *Podospora* (7.5% of OTUs) and *Sporormiella* (3.0% of OTUs)^113^, the spores of which are ingested by the grazing herbivores and subsequently germinate in dung, so that they are also used as indices of variation through time of large herbivore populations^114^. All together, these taxa comprised more than 25% of the OTUs. Belanche et al.^48^ observed that pathogen and saprotroph fungi increased in the rumen of sheep moved to pasture after a diet of hay and concentrate, although their relative abundance remained lower than that observed in our study. We suggest that the longer period at pasture experienced by the cows of this study is the reason for the high presence of ingested fungi in the faeces. In any case, the results of our study suggest that the gut fungal community may be strongly influenced by grazing, because of ingested taxa that increase at the expense of gut-symbiont species, and further studies are needed to elucidate these hypotheses. Also we suggest that this intake of fungi with the forage was the main reason for the time-related variability of the fungal community, with a seasonal dynamics similar to that observed for plants. In fact, none of the Neocallimastigaceae showed temporal variations in the mixed linear regression models, while significant differences were found for Ascomycota and Basidiomycota, among which *Camarosporium* spp. increased from 5–7% in T1 and T2 to more than 10% of total OTUs in the second half of the summer, and *Ustilago striiformis* from close to 0 to almost 3%. This might also indicate that the fungal taxa that interact with the microbial gut ecosystem to ensure the fermentation of cell walls remained constant during the summer. This is understandable because, irrespective of the changes in botanical composition, the pasture-based diet remained characterized by a high cell-wall content.

In contrast with plants, the fungal community showed a certain degree of breed specificity, although limited to few species which were always more abundant in the Simmental. These included taxa with highest similarities to *Preussia* spp. (*p* < 0.001), *Preussia flanaganii* (*p* < 0.001), which are known as dung fungi^115^, and an unidentified member of the Agaricales (*p* < 0.001), which are mushroom fungi^115^. Therefore, we argue that also the differences between breeds did not reflect a different symbiont fungal community, but were instead the result of differences in ingested taxa that might be due to the higher number of samples of the Simmental cows.

The prevailing bacteria were found to belong to the Ruminococcaceae family which are obligate anaerobes and typical inhabitants of the gut tracts of mammals^116^. Within the family Rikenellaceae (Phylum Bacteroidetes), which is known for its contribution to gut metabolomics^117^, particularly abundant was the presence of the genus *Alistipes*. A certain presence of the Archaea was also observed: this kingdom was essentially represented by the genus *Methanobrevibacter* (family Methanobacteriaceae), a strict anaerobe that can produce methane by reduction of carbon dioxide via hydrogen^118^. Overall, our results are in agreement with the range of bacterial taxa found in literature^65,119,120^, and more specifically the prevalence that we found for Firmicutes and Bacteroidetes is in line with that observed by others on fecal cattle microbiota^49,67,121^, even in widely different dietary and health conditions. These results indicate that the temporal variations in diet quality and external factors that might influence gut microbiota, as for instance temperature^122^ were not sufficient to induce appreciable variations in the bacterial microbiome, as was conversely observed in a different study^54^ with wider dietary and seasonal conditions. This finding was not surprising, since even much more marked dietary treatments, as shifts from pasture-based to hay-concentrate or total mixed diets, did not show remarkable changes in the main bacterial taxa^48,123^. Instead, for a given animal host species maintaining a constancy in the gut bacteriome is a physiology-associated endeavor, which can be so constrained that even breed-level specificities can be featured^64–66^, as displayed in our results. We suggest therefore that in future studies the microbiome stability in grazing ruminants should be further examined considering the variability of external factors in relation to host factors, as breed and/or individual features but also stressful conditions that are not uncommon in grazing conditions^124–126^. Interestingly, when the breed variable was considered, the three communities (plants, fungi and bacteria) displayed the opposite scenario: bacteria appeared to be the most clearly separated group, and their ordination between breeds had the highest significance levels; fungi showed also a degree of cattle breed-specificity, but lower than bacteria, and the estimated plant diet composition appeared to be substantially the same for the two breed that shared the same foraging areas. In this respect a major role is likely displayed by the stable core microbiome, whereby more transient microbial taxa would contribute to the gut adaptation to diet changes^45^.

In terms of results consistency as a function of data processing, in addition to the square root transformation we tested also the Aitchison’s centered log-ratio transformation (CLR), which is deemed as more appropriate to cope with the issue of the compositional nature of the datasets^127^. Apart from some slight changes in the shape of the ordination plots, the observed phenomena and the ensuing trends were found to be exactly the same for both transformations. We opted for showing the square root transformation because its combination of total sum scaling is the Hellinger transformation which has been praised as a preferable choice in ecological community comparisons^128^, as it offers the best trade-off between linearity and resolution in comparison to chi-square metrics and other approaches. It is also recognized as more balanced for the weight given to rare species, and for this reason it is the first recommended choice in the Calypso webtool suite^81^.

**Figure 5:**
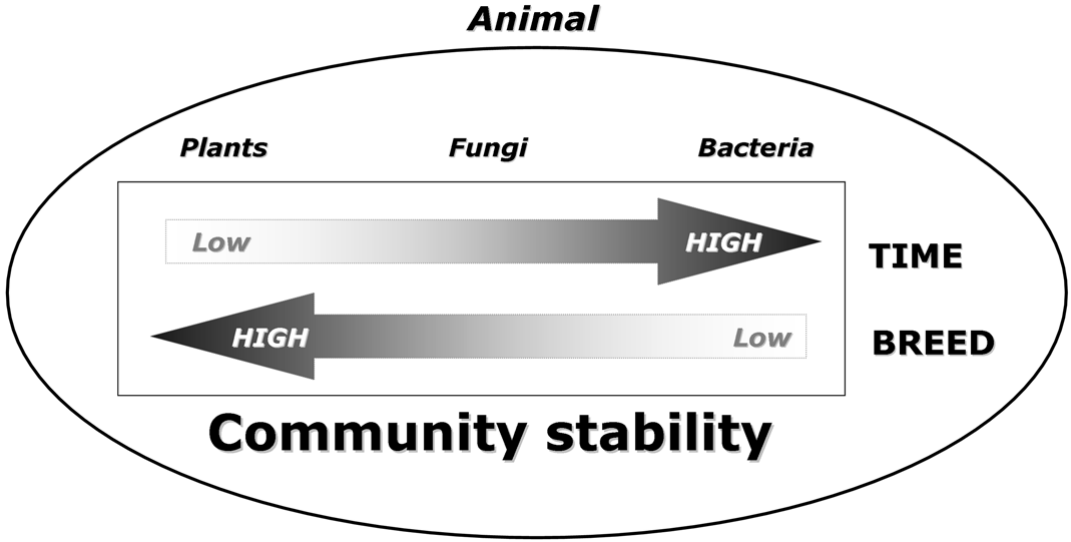
Depiction of the observed trends outlining the reciprocal gradients of community stability versus variation as a function of the two inspected variables of sampling time and cow breed. The pattern of plant diet composition (unstable in time, since ruled by their intra-seasonal availability succession but showing no preference in consumption by the two breeds) is opposite to that of bacteria (stable as gut residents irrespective of the seasonally changing botanical composition of the diet but displaying breed-specific composition). The fungal component, belonging partly to the grazed transient food and partly to the cow microbiota, showed an intermediate pattern. The fourth component is the Animal host kingdom, within which the above dynamics appear to unfold.

In essence, in this multi-kingdom survey from bovine faecal material targeting, at the same time, the plant diet composition and the two corporations of the gut microbiota, we have analysed three biotic communities (summarized in Fig. 5), spanning from plants to fungi and bacteria. The time factor showed a decreasing gradient of successional dynamics from plants to bacteria. Conversely, when the host breed was considered, the three kingdoms showed the opposite gradient of variation, where bacteria resulted the ones mostly characterized by host-specificity. In other words, plants varied with time while being practically non-breed specific, because it was apparently their own seasonal availability to impose the constraint to use them as available diet. The two breeds, in turn, did not show differences in plant selectivity, while bacteria, that were apparently influenced by host but not by diet, showed breed specificity but no variation with time: Finally, fungi, which were determined both by host factors and by intake with diet, were in both cases found in an intermediate position between plants and bacteria. While these patterns could have been perhaps hypothesized to occur for plants and bacteria, they were hitherto unknown for fungi. Therefore, comparing these three levels allowed to show how the gradients of effects exerted by time and breed variables varied across the groups of organisms analysed.

In conclusion, this study adds new knowledge, at more detailed spatial and temporal scales than those available from the very few studies conducted in markedly different conditions, on diet selection and covariation of gut microbiota for domestic ruminants grazing semi-natural grasslands. It is presumably because of this and of the different habitat conditions, that our results are different from those previously available, and can be considered relevant for high elevation pastures that are widely grazed across European mountain regions. We stress the importance that the potential of DNA metabarcoding should be further exploited in the future to address the gap in knowledge on plant-host-microbiota interactions that is necessary to fill in order to manage and conserve the grassland ecosystems. This study, connecting the four corners of animals, plants, fungi and bacteria in a coherent physiological chain, offers novel insights in the reciprocal interactions on which the different players of an ecosystem base their trophic relationships and structure their dynamic living networks.

## Supplementary Information

Supplementary Figures.

Supplementary Tables.

## Data Availability

The data that support the findings of this study are available from the corresponding author upon request. All data regarding taxa identity and abundance for plants, fungi and bacteria are shown in the Supplementary tables dataset.
