# Supplementary material for: A multi-kingdom metabarcoding study on cattle grazing Alpine pastures discloses intra-seasonal shifts in plant selection and faecal microbiota
Source: Sci Rep. 2021 Jan 13;11:889. doi: 10.1038/s41598-020-79474-w (PMC7806629; doi:10.1038/s41598-020-79474-w)
Supplement: Supplementary file 1 — Supplementary Figures. [file 41598_2020_79474_MOESM1_ESM.pdf]

Supplementary Figures

A multi-kingdom metabarcoding study on cattle grazing Alpine pastures discloses intra-seasonal shifts in plant selection and faecal microbiota

Fabio Palumbo[1], Andrea Squartini[1*], Gianni Barcaccia[1], Stefano Macolino[1], Cristina Pornaro[1], Massimo Pindo[2], Enrico Sturaro[1], Maurizio Ramanzin[1]

[1]Department of Agronomy Food Natural Resources Animals and Environment (DAFNAE), University of Padova, Campus of Agripolis, Viale dell'Università 16, 35020 Legnaro, Padova, Italy

[2]Research and Innovation Centre, Fondazione Edmund Mach (FEM), Via Mach 1, 38010 S. Michele all'Adige, Trento, Italy

[*]Corresponding author: Andrea Squartini - squart@unipd.it

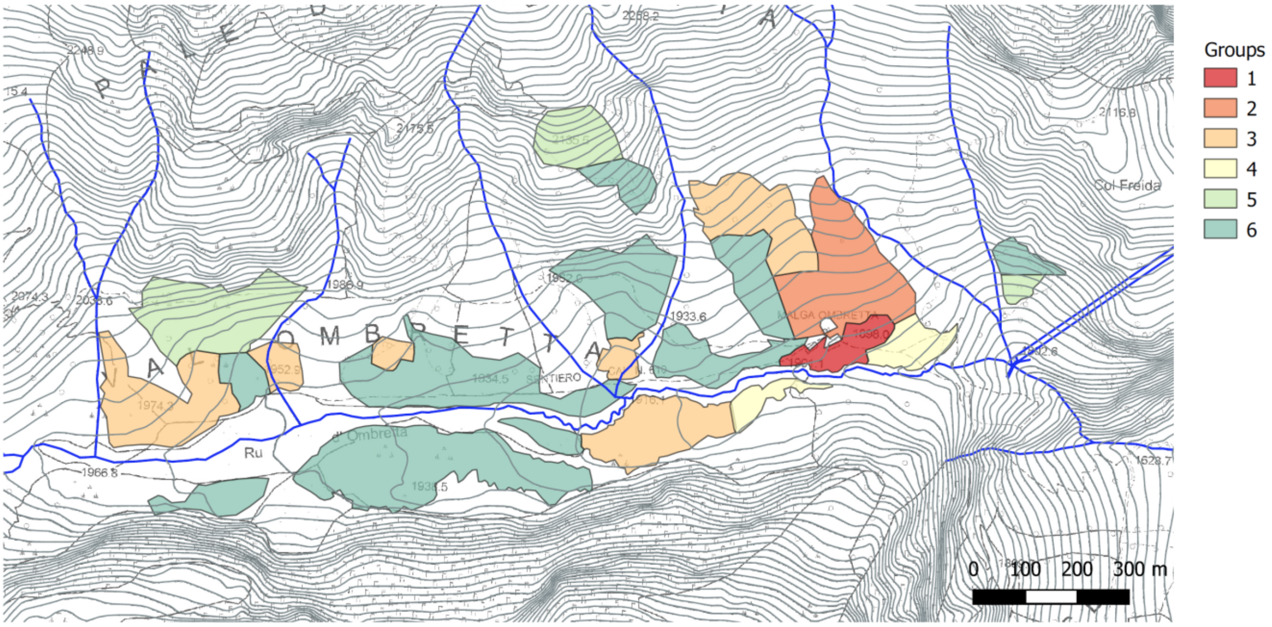

Supplementary Figure S1. Spatial distribution of groups resulting from the cluster analysis of the species matrix obtained from botanical surveys performed in the grazing surface of "Ombretta". This map was generated with QGIS software v.2.12 (QGIS Development Team, available at http://qgis.osgeo.org).

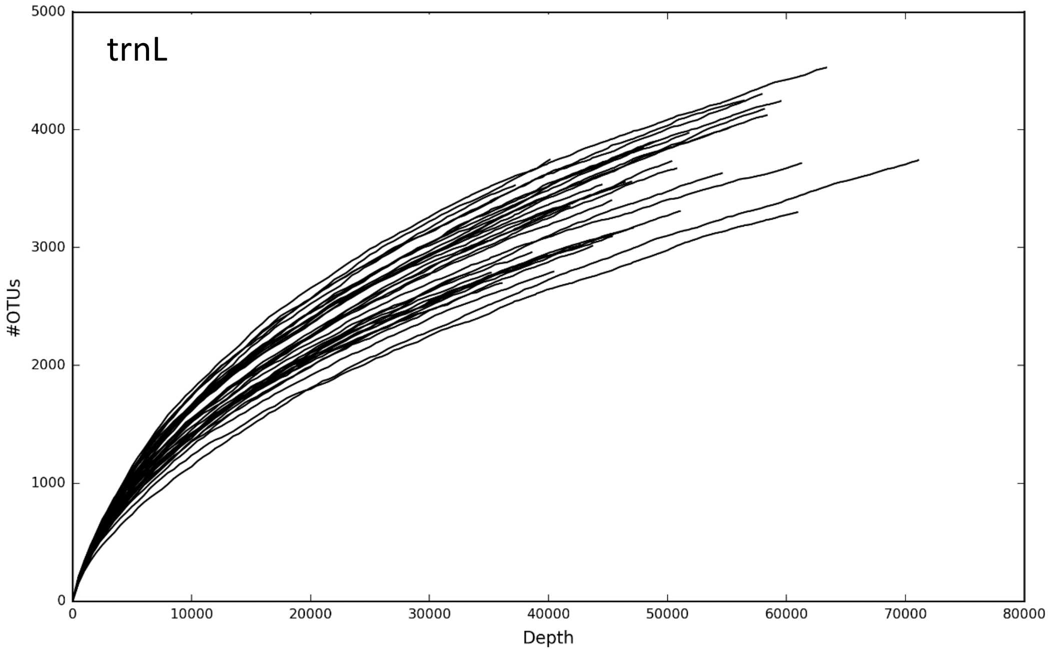

Supplementary Figure S2. Rarefaction curves comparing the number of reads (depth) with the number of plant OTUs (Eukaryota domain, Plant kingdom) detected in the gDNA extracted from 32 faecal samples.

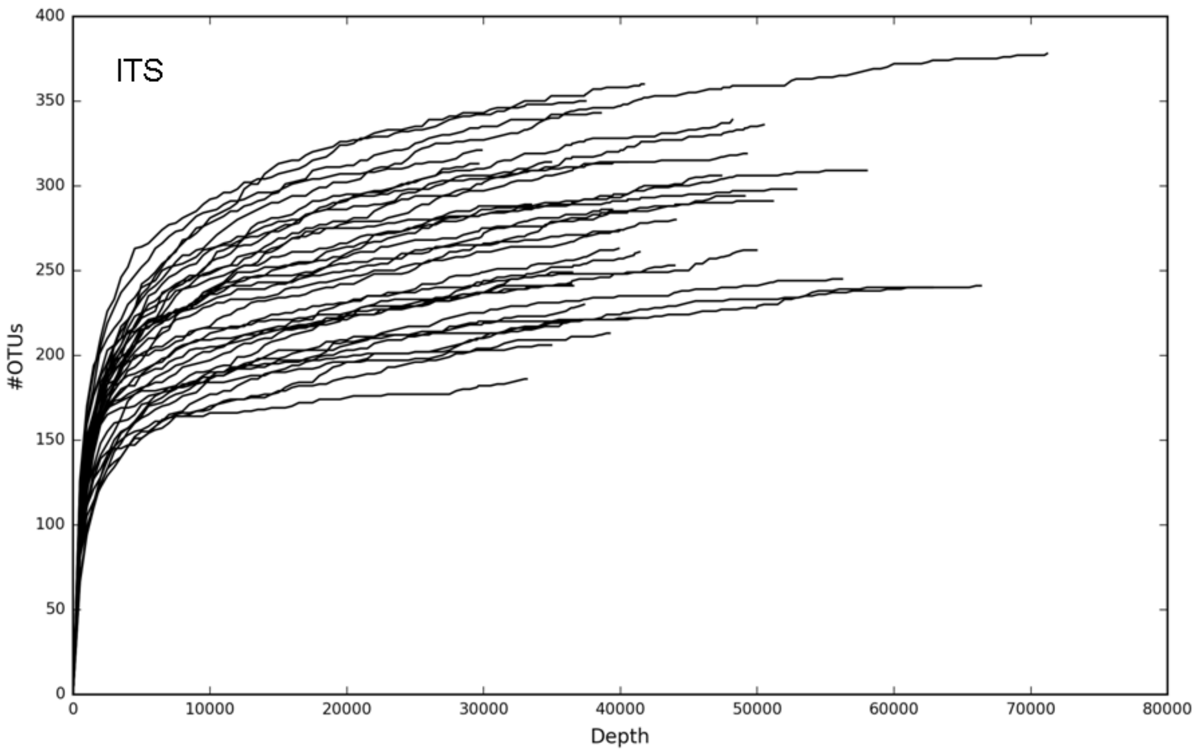

Supplementary Figure S3. Rarefaction curves comparing the number of reads (depth) with the number of fungal OTUs (Eukaryota domain, Fungi kingdom,) detected in the gDNA extracted from 32 faecal samples.

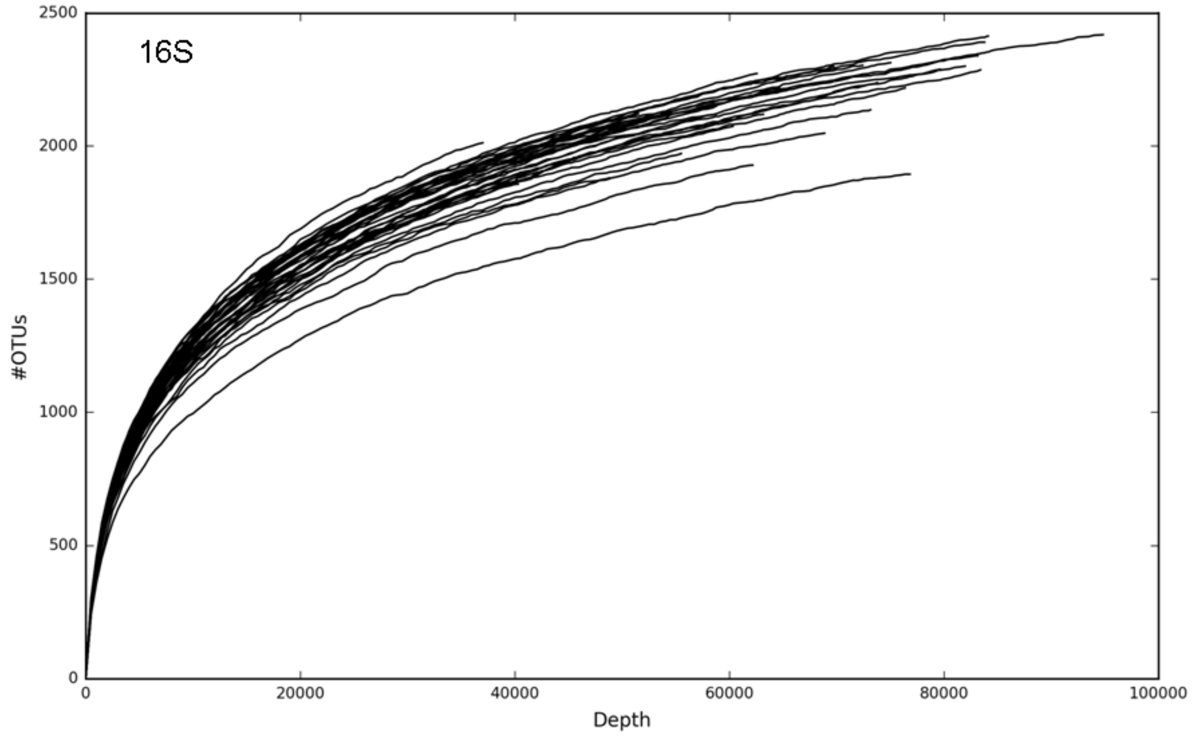

Supplementary Figure S4. Rarefaction curves comparing the number of reads (depth) with the number of bacterial OTUs (Prokaryota domain, Bacteria and Archea kingdoms) detected in the gDNA extracted from 32 faecal samples.

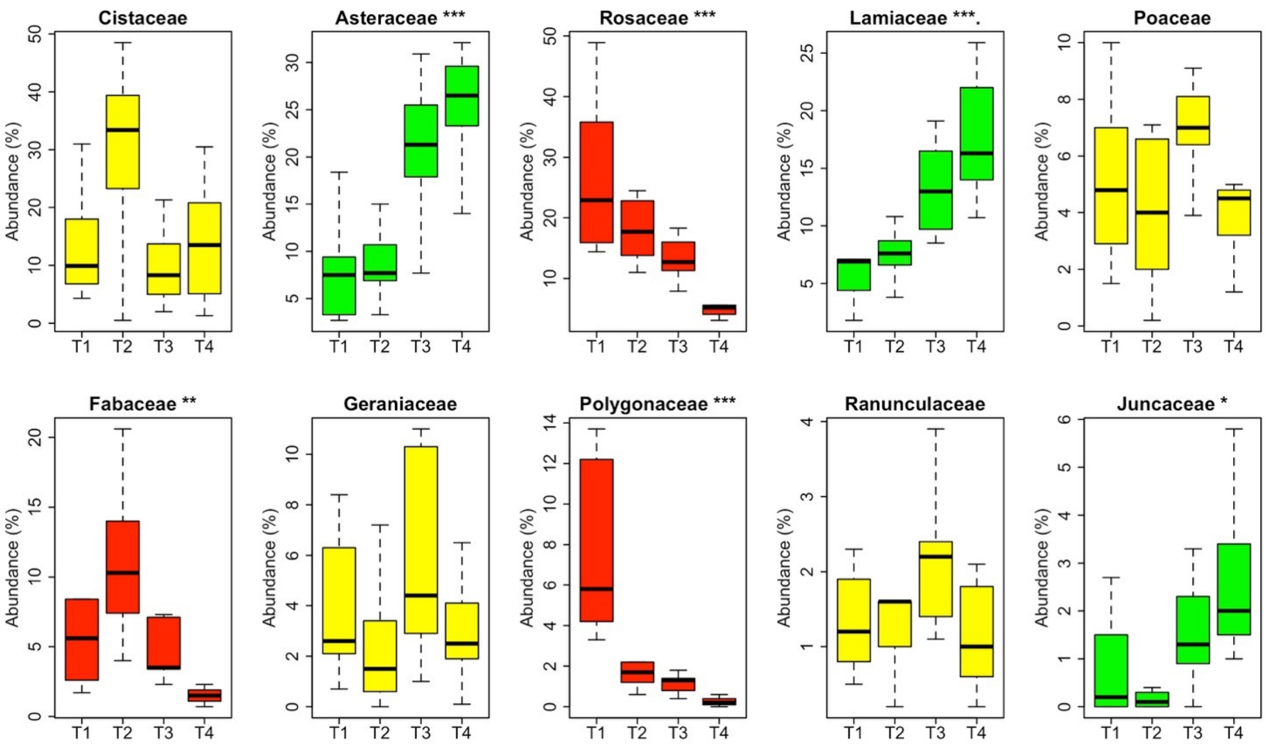

Supplementary Figure S5. Variation of the relative abundance (% of total abundance) of the 10 most abundant plant families in relation with collection time (T1, T2, T3, T4). Plant families are ordered from left to right and top to bottom in order of decreasing relative abundance. The significance of the effect of collection time in the linear mixed effect regression models analyzing relative abundances (standardized with total sum scaling (TSS) normalization followed by square route transformation) is indicated as ***: P<0.001; **: P<0.01; *: P<0.05. Boxplots in yellow indicate no significant temporal trends, boxplots in green indicate a pattern of increase with collection time, boxplots in red indicate a pattern of decrease.

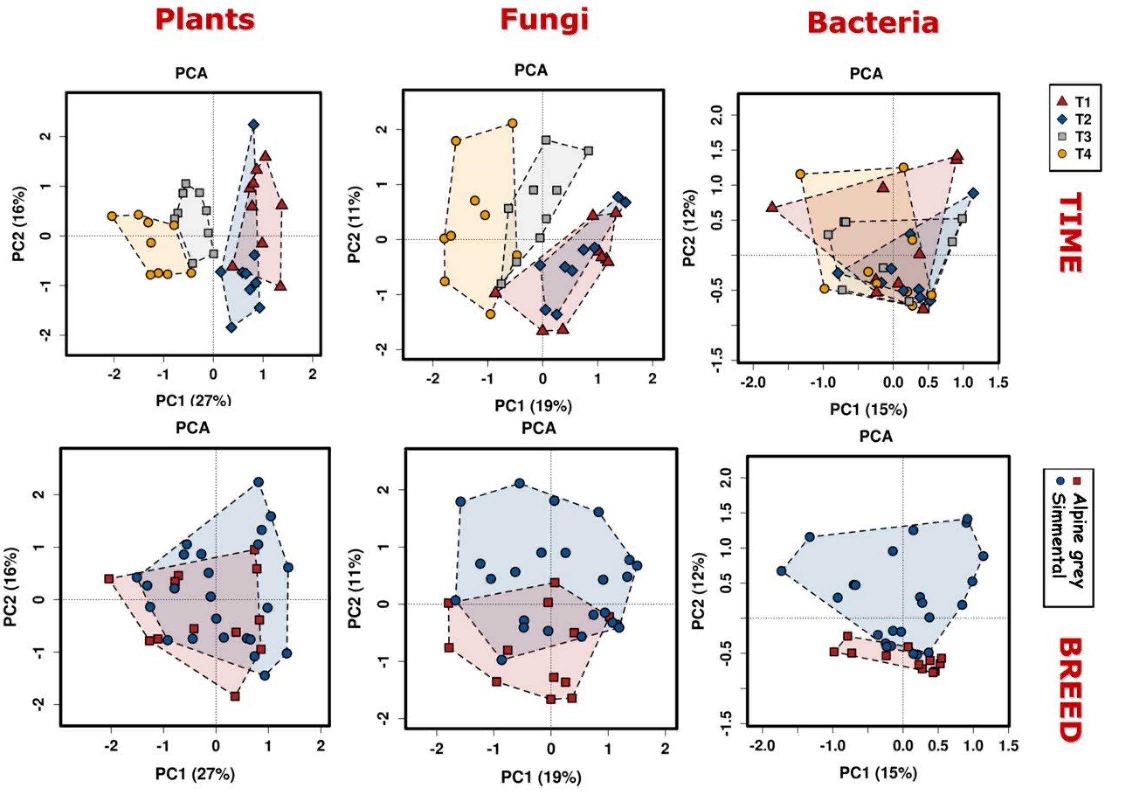

Supplementary Figure S6. Principal component analysis (PCA) showing synoptically the ordination plots of plant, fungal and bacterial taxa for the two variables of sampling time and cow breed.

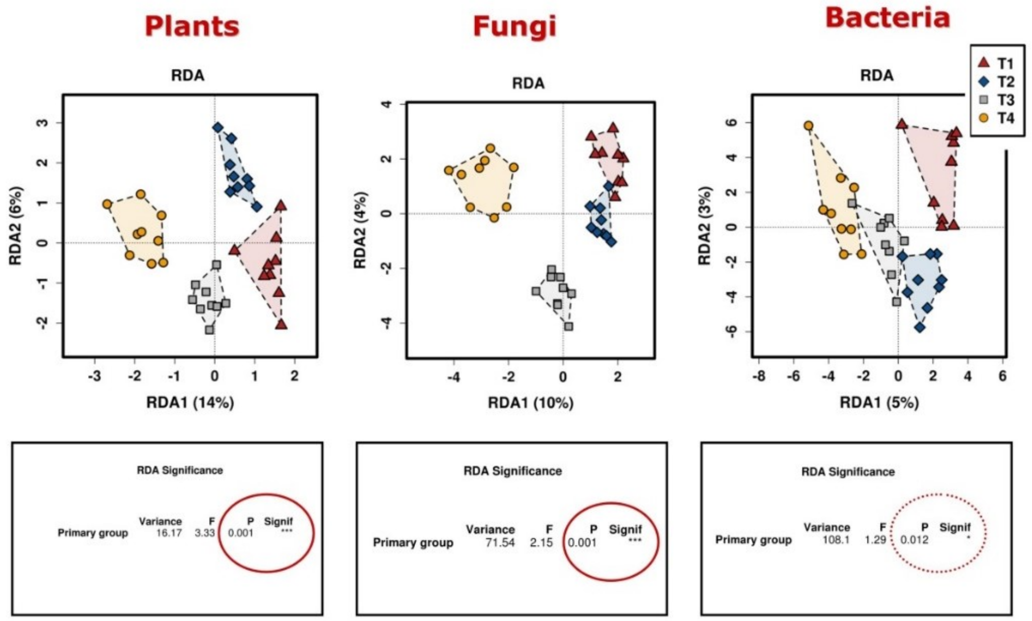

Supplementary Figure S7. Redundancy Analysis showing synoptically the ordination plots of plant, fungal and bacterial taxa for the sampling time variable. The respective significance levels are showed below and circled in red when significant p values result.

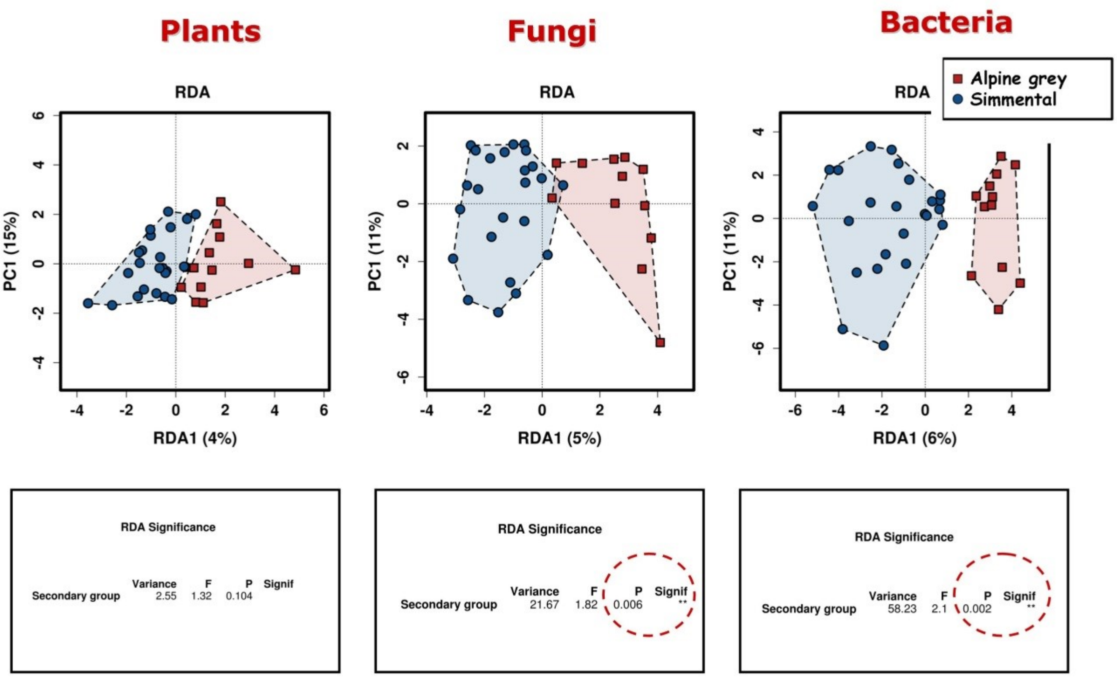

Supplementary Figure S8. Redundancy Analysis showing synoptically the ordination plots of plant, fungal and bacterial taxa for the breed variable. The respective significance levels are showed below and circled in red when significant p values result.

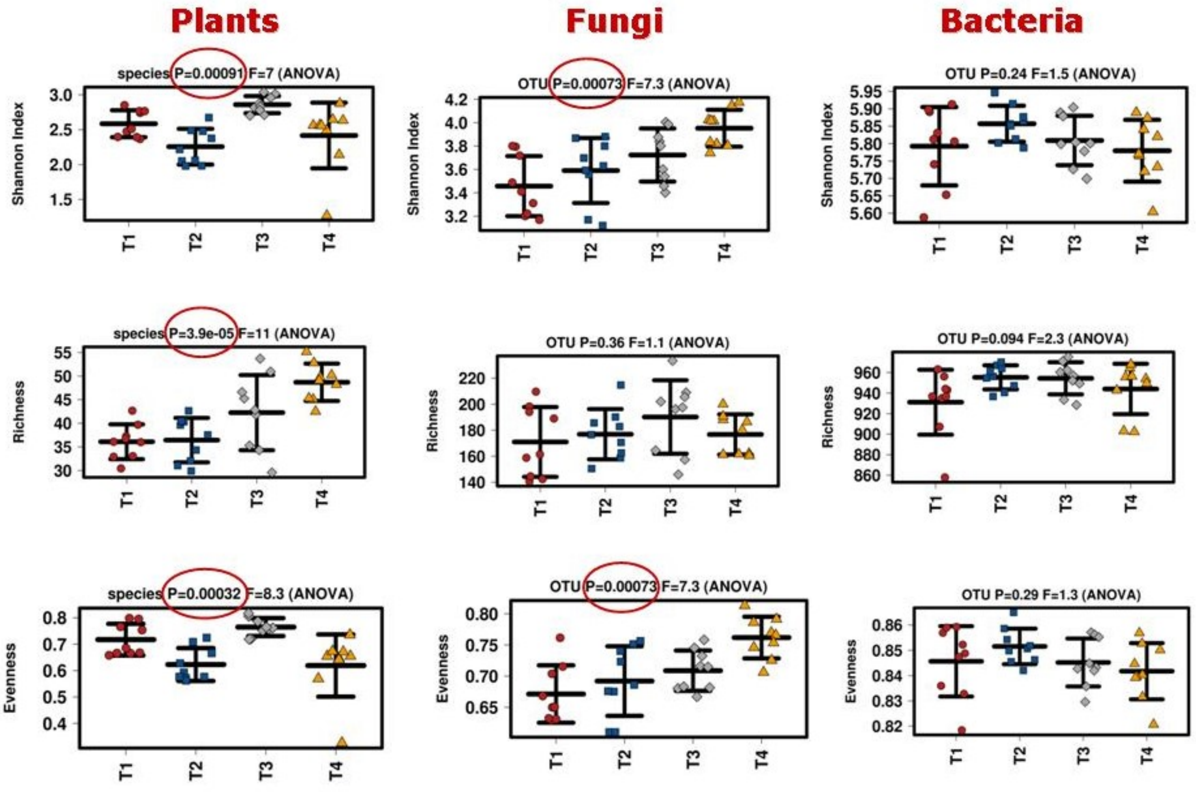

Supplementary Figure S9. Ecological indexes of the plant, fungal and bacterial communities occurring within the bovine faeces in relation to sampling time. Significance levels of the comparison are showed above and circled in red when significant p-values result.

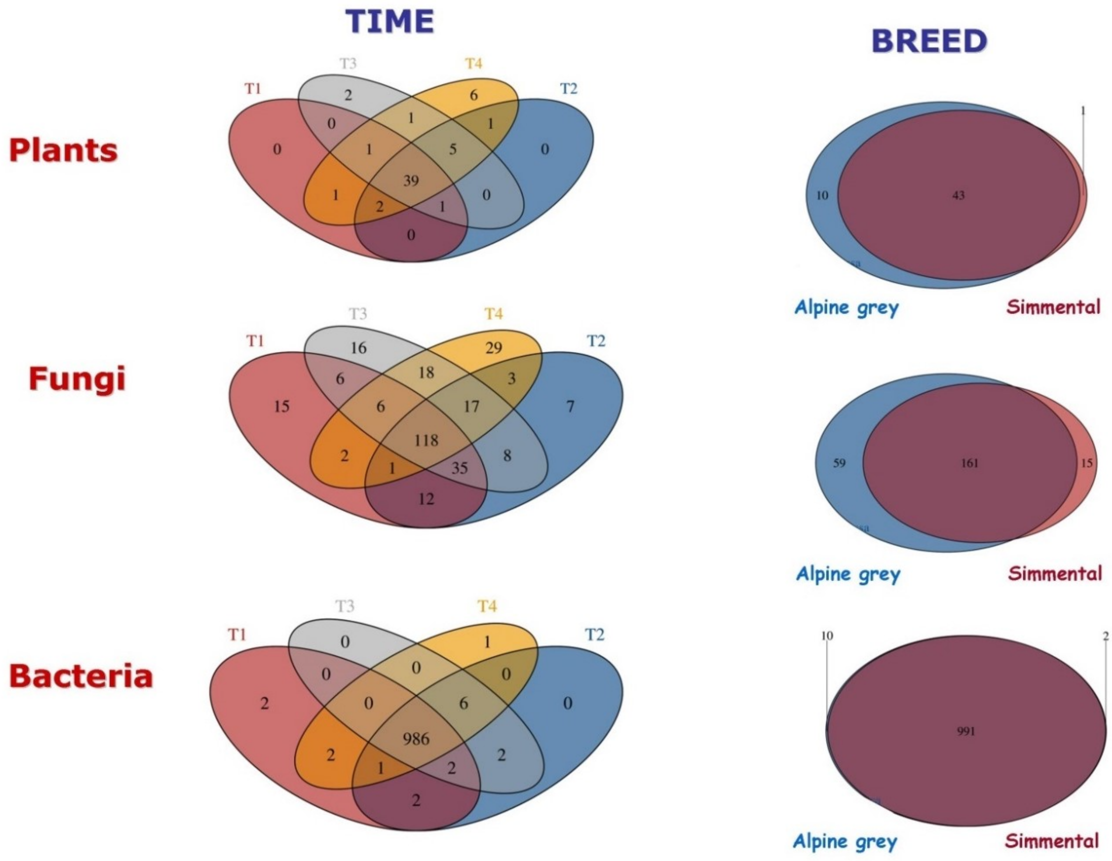

Supplementary Figure S10. Venn diagrams of shared and specific taxa found in the three analyses, according to collection time and breed.

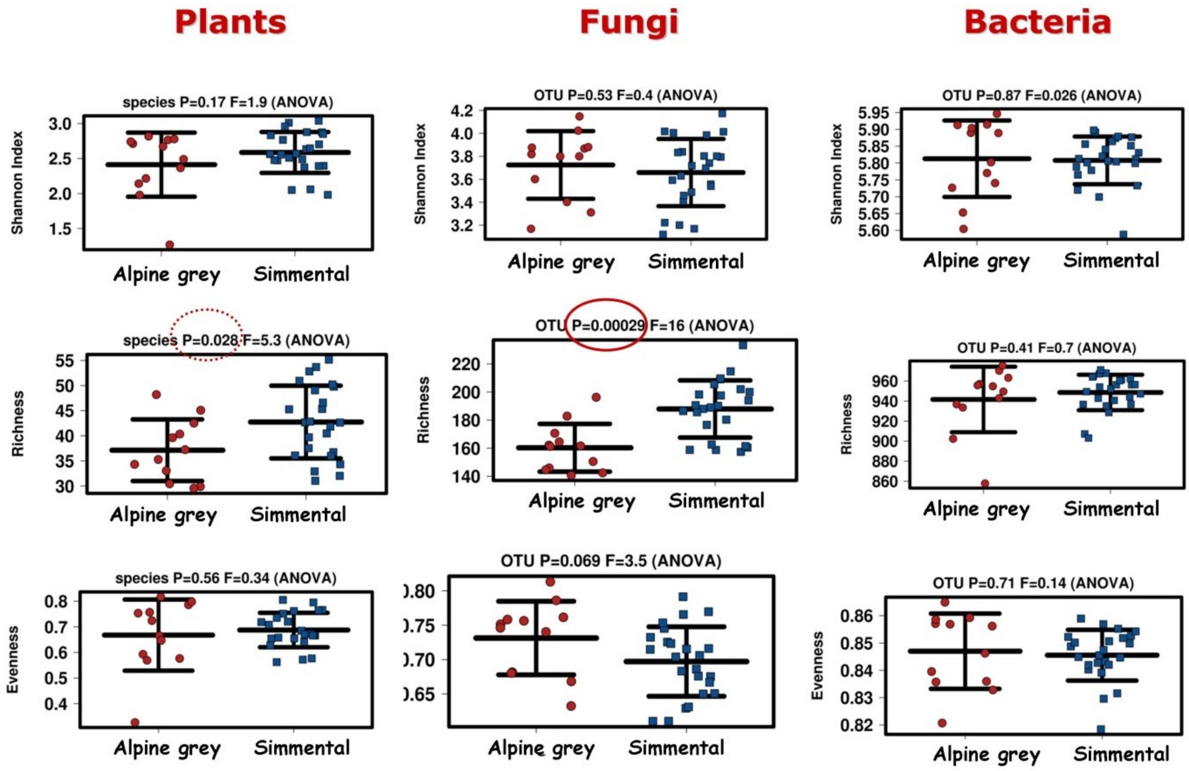

Supplementary Figure S11. Ecological indexes of the plant, fungal and bacterial communities occurring within the bovine faeces in relation to cow breed. Significance levels of the comparison are showed above and circled in red when significant p values result.

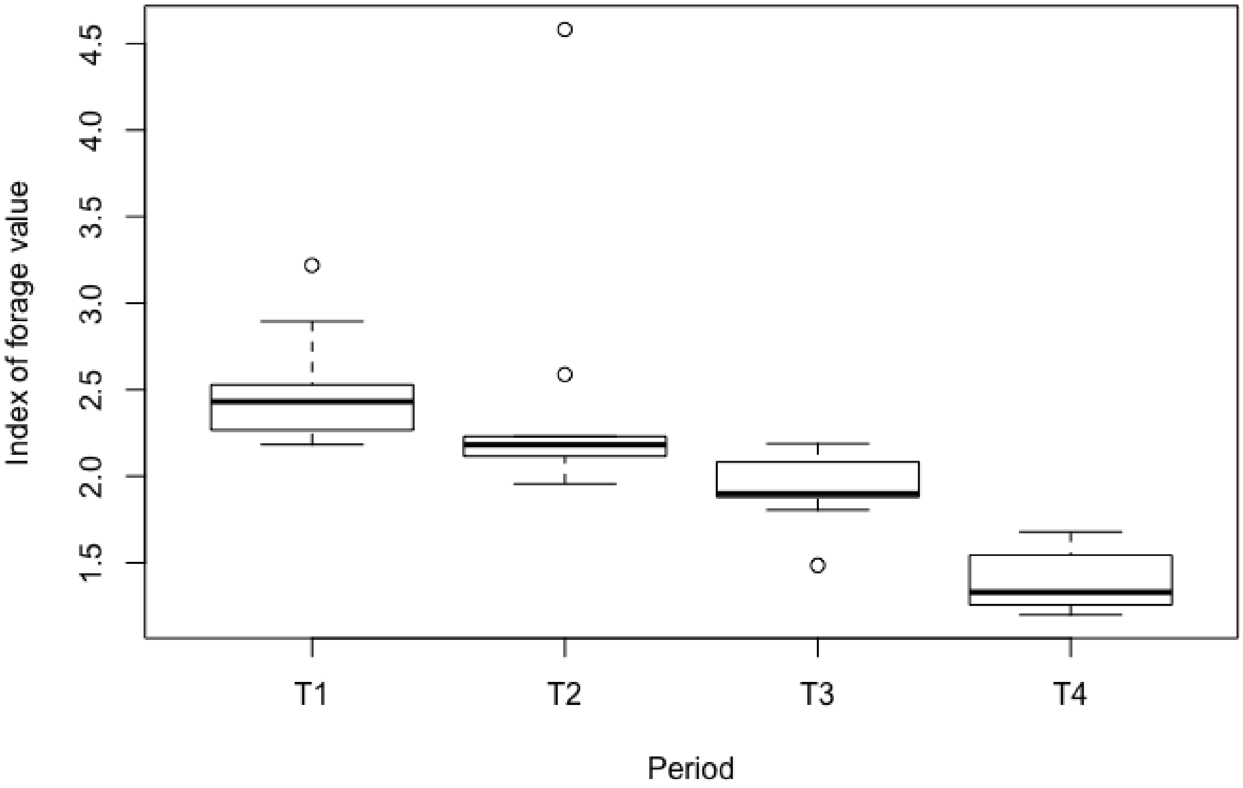

Supplementary Figure S12. Temporal trend of the weighted "forage values" calculated for each faecal sample (see main text for details). According to linear mixed modelling of log-transformed values, differences are not significant between T1 and T2, while are significant at $P<0.01$ for T3 and at $P<0.01$ for T4. Non-transformed data are shown for better clarity

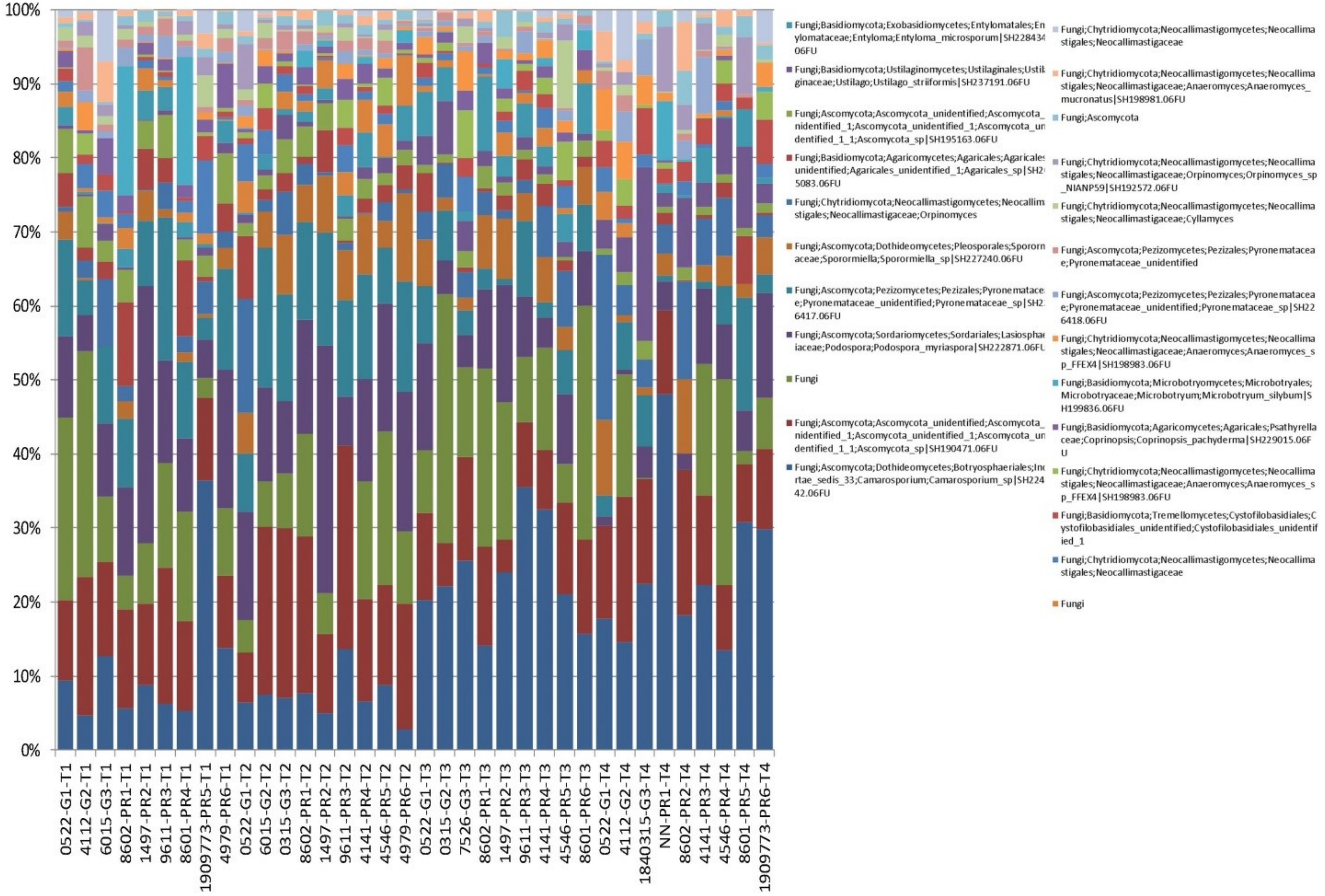

Supplementary Figure S13. 25 most abundant fungal OTUs identified.

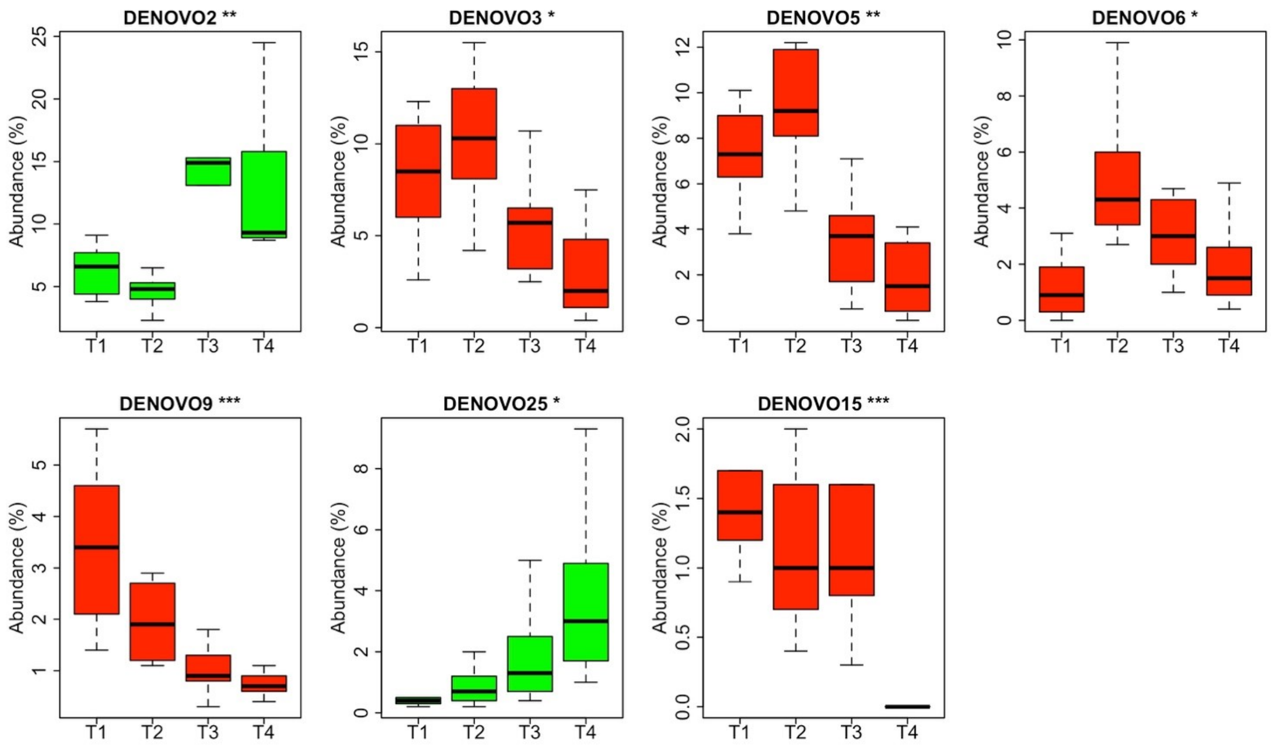

Supplementary Figure S14. Relative abundance (% of total abundance) of the fungal taxa that varied significantly in relation with collection time (T1, T2, T3, T4) among the 25 most abundant taxa. Taxa are ordered from left to right and top to bottom in order of decreasing relative abundance. Only identified taxa are shown. The significance of the effect of collection time in the linear mixed regression models analyzing relative abundances (standardized with total sum scaling (TSS) normalization followed by square route transformation) is indicated as ***: $P<0.001$; **: $P<0.01$; *: $P<0.05$. Boxplots in green indicate an increase with collection time, boxplots in red a decrease.

OTUs are identified as:

- DENOVO2: Ascomycota; Dothideomycetes; Botryosphaeriales; Incertae sedis 33; Camarosporium; Camarosporium sp|SH224142.06FU.
- DENOVO3: Ascomycota; Sordariomycetes; Sordariales; Lasiosphaeriaceae; Podospora; Podospora_myriaspora|SH222871.06FU
- DENOVO5: Ascomycota; Pezizomycetes; Pezizales; Pyronemataceae; Pyronemataceae unidentified; Pyronemataceae sp|SH226417.06FU
- DENOVO6: Ascomycota; Dothideomycetes; Pleosporales; Sporormiaceae; Sporormiella; Sporormiella sp|SH227240.06FU
- DENOVO9: Ascomycota; Ascomycota unidentified; Ascomycota unidentified 1; Ascomycota unidentified 1; Ascomycota unidentified 1_1; Ascomycotasp|SH195163.06FU
- DENOVO25: Basidiomycota; Ustilaginomycetes; Ustilaginales; Ustilaginaceae; Ustilago; Ustilago striiformis|SH237191.06FU
- DENOVO15: Basidiomycota; Agaricomycetes; Agaricales; Psathyrellaceae; Coprinopsis; Coprinopsis pachyderma|SH229015.06FU

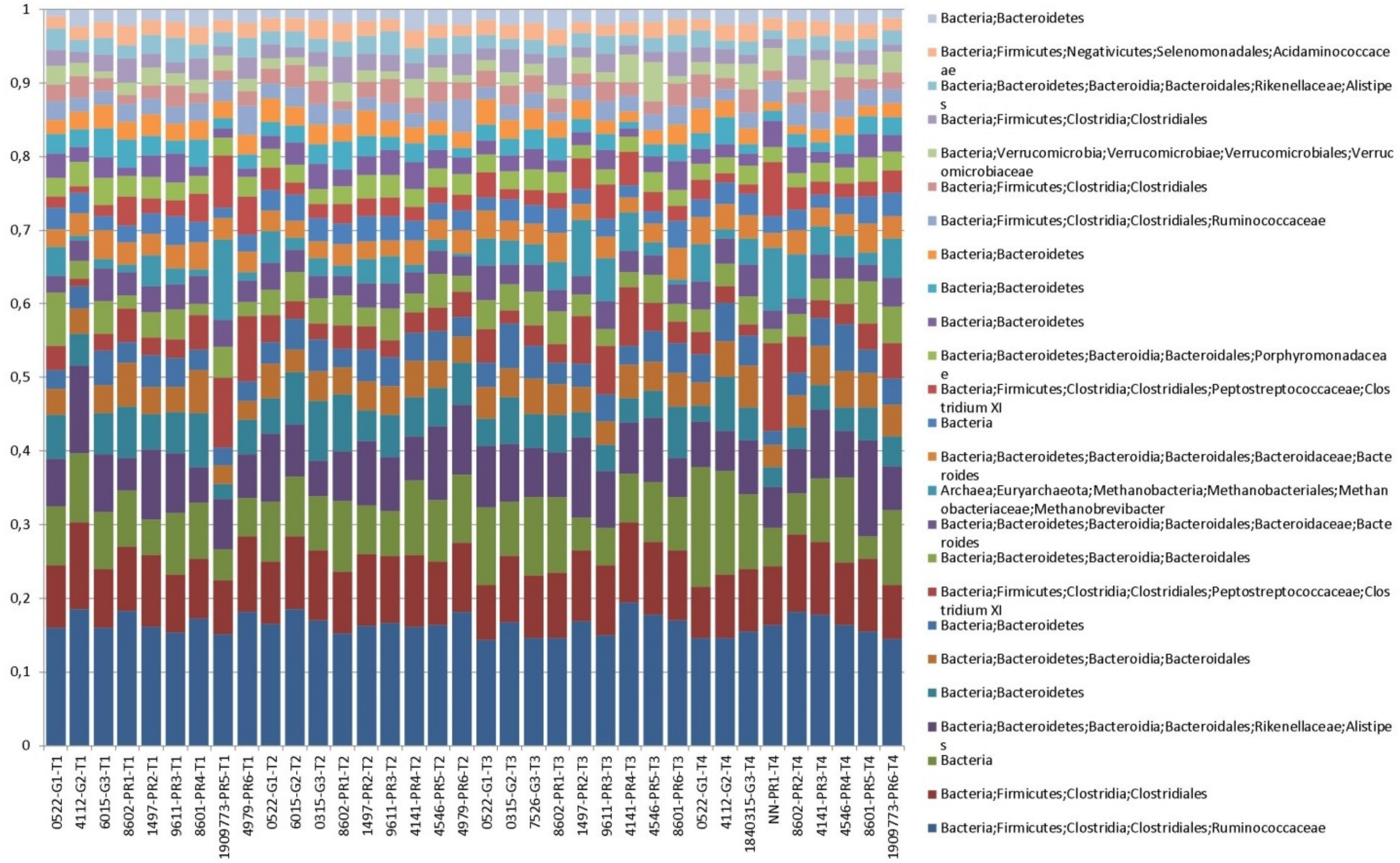

Supplementary Figure S15. 25 most abundant bacterial OTUs identified.
